# Insertion and deletion evolution reflects antibiotics selection pressure in a Mycobacterium tuberculosis outbreak

**DOI:** 10.1371/journal.ppat.1008357

**Published:** 2020-09-30

**Authors:** Maxime Godfroid, Tal Dagan, Matthias Merker, Thomas A. Kohl, Roland Diel, Florian P. Maurer, Stefan Niemann, Anne Kupczok

**Affiliations:** 1 Institute of General Microbiology, Kiel University, Kiel, Germany; 2 Molecular and Experimental Mycobacteriology, Research Center Borstel, Borstel, Germany; 3 German Center for Infection Research, Partner Site Hamburg-Lübeck-Borstel-Riems, Borstel, Germany; 4 Institute for Epidemiology, University Medical Hospital Schleswig-Holstein, Kiel, Germany; 5 Lungenclinic Grosshansdorf, Airway Research Center North (ARCN), Member of the German Center for Lung Research (DZL), Großhansdorf, Germany; 6 National and WHO Supranational Reference Center for Mycobacteria, Research Center Borstel, Borstel, Germany; 7 Institute of Medical Microbiology, Virology and Hygiene, University Medical Center Hamburg-Eppendorf, Hamburg, Germany; McGill University, CANADA

## Abstract

In genome evolution, genetic variants are the source of diversity, which natural selection acts upon. Treatment of human tuberculosis (TB) induces a strong selection pressure for the emergence of antibiotic resistance-conferring variants in the infecting *Mycobacterium tuberculosis* (MTB) strains. MTB evolution in response to treatment has been intensively studied and mainly attributed to point substitutions. However, the frequency and contribution of insertions and deletions (indels) to MTB genome evolution remains poorly understood. Here, we analyzed a multi-drug resistant MTB outbreak for the presence of high-quality indels and substitutions. We find that indels are significantly enriched in genes conferring antibiotic resistance. Furthermore, we show that indels are inherited during the outbreak and follow a molecular clock with an evolutionary rate of 5.37e-9 indels/site/year, which is 23 times lower than the substitution rate. Inherited indels may co-occur with substitutions in genes along related biological pathways; examples are iron storage and resistance to second-line antibiotics. This suggests that epistatic interactions between indels and substitutions affect antibiotic resistance and compensatory evolution in MTB.

## Author summary

*Mycobacterium tuberculosis* (MTB) is a human pathogen causing millions of deaths every year. Its genome evolution has been intensively characterized through point substitutions, i.e., nucleotide exchanges that are inherited. Additional mutations are insertions and deletions of nucleotides, termed indels, that can involve few nucleotides up to thousands and even more. Short indels in genes might change the reading frame and disrupt the gene product. Here we show that indels occur frequently in genes causing antibiotic resistance upon disruption suggesting that antibiotic treatment has a strong impact on indel evolution in an MTB outbreak. Furthermore, we show that the molecular clock, i.e., the temporal emergence of variants over time, holds for short indels in MTB genomes. Finally, we observe that indels may co-occur with substitutions in genes along related biological pathways. These results support the notion that indels are important contributors to MTB evolution. We anticipate that including indels in the analyses of MTB outbreaks will improve our understanding of antibiotic resistance evolution.

## Introduction

*Mycobacterium tuberculosis* complex (MTBC) strains, the causative agents of tuberculosis (TB), are strict host-associated pathogens [1]. With estimated numbers of ten million new infections and 1.2 million deaths in 2018 [2], TB is a major cause of human disease and mortality. In addition, *Mycobacterium tuberculosis sensu stricto* (MTB), the human-adapted member of the MTBC, shows a high level of intrinsic and evolved antibiotic resistance (ABR), including multi-drug resistance (MDR) [3]. MTB genomes have a low genetic diversity and furthermore, comparative genomics of MTB genomes showed that genetic variation is only vertically inherited, likely due to the absence of horizontal transfer mechanisms in MTB [4,5]. Consequently, MTB antibiotic resistance is considered to evolve *de novo* via point and segmental mutations and not by horizontal transfer of genetic material [6]. Antibiotic resistance may induce high fitness costs that are frequently ameliorated by compensatory mutations [7]. For example in MTB, mutations in *rpoB*, encoding the beta-subunit of the RNA polymerase, can lead to rifampicin resistance [8] and mutations in *rpoC* often compensate ABR-conferring mutations in *rpoB* [9,10]. Notably, in asexual organisms, beneficial alleles are linked to the genetic background where they appeared. This results in competition between beneficial alleles (also known as clonal interference) and the hitchhiking of neutral or slightly deleterious alleles with beneficial ones. Indeed, time series patient sampling revealed that clonal interference and hitchhiking contribute to antibiotic resistance evolution in MTB [11,12].

Genetic variation in MTB strains is generally characterized by substitutions that are observed as single-nucleotide polymorphisms (SNPs). Substitutions are the major source of variation in MTB genomes followed by insertions and deletions (hereafter denoted as indels) [13]. Short indels (up to 50 bp) were found to occur primarily in non-coding regions, in the repeat-containing PE-PPE genes and in ABR-conferring genes [13]. Additionally, long insertions in MTB are mainly due to integration of the mobile element IS6110, a transposase-mediated insertion sequence [14]. Importantly, previous studies analyzing MTB strain genome evolution provided evidence for the role of indels in ABR evolution [15–17]. Some well-known examples of indels causing antibiotic resistance are the deletion of *katG* conferring isoniazid resistance [18] and the disruption of *pncA* conferring pyrazinamide resistance [19].

Similarly to resistance determination, the evolutionary relationships of MTB strains that have been sampled from outbreaks are generally inferred by SNP-based phylogeny reconstruction. To this end, SNPs are detected from short-read sequencing data that is aligned to the complete and well characterized reference genome H37Rv [20,21]. Outbreak reconstructions have furthermore been used to identify signals of positive selection in MTB strain evolution, for example, by identifying convergent evolution, i.e., variants that evolved independently multiple times. Convergent evolution in MTB has been observed in ABR-conferring genes [22] and in virulence factors [23]. Furthermore, time-series sampling of MTB strains showed that substitutions in MTB genomes evolve at an approximately constant pace, i.e., substitutions follow a molecular clock [24]. Notably, the substitution rate of MTB is at the lower end of the spectrum of prokaryotic substitution rates [6]. Despite the low evolutionary rate observed for MTB strains, molecular dating can be used to infer the time of emergence of ABR-conferring substitutions [25] and the introduction time of strains into specific parts of the world [26].

Although previous studies extensively investigated the rate and impact of substitutions in MTB strain evolution, the frequency and contribution of indels to MTB evolution in outbreak settings has been sparsely analyzed. To address this question, we estimated the evolutionary rate and the putative phenotypic impact of insertions and deletions in MTB outbreak strains. As a paramount example for drug resistance evolution, we analyze a previously described multi-drug resistant clade of 353 MTB lineage 2 (Beijing) strains, i.e., the Central Asian outbreak (CAO) [27]. We further compare some aspects of indel evolution to the drug-susceptible lineage 4 (Euro-American) ‘Hamburg outbreak’, where 64 strains have been sequenced [28].

## Results

### Genome-wide indels in the CAO and in the Hamburg outbreak are detected by high-quality variant inference

To study the evolution of point and segmental mutations in *M*. *tuberculosis*, we analyzed 353 MTB strains of the lineage 2 CAO isolated between 1995 and 2015 (S1A Table). The criteria for strain inclusion in the dataset were the presence of genetic markers defining the CAO, which are known to be involved in transmission of multi-drug resistant TB mainly in central Asia [27]. The strain collection was mainly assembled from a previously published collection derived from a drug resistance survey in Karakalpakstan, Uzbekistan [10] and from routine MDR-TB surveillance data from German patients with 199 newly generated datasets. Genetic variants were inferred by comparing the sample genomes to a closely related reference genome (strain *M*. *tuberculosis* 49–02 [27]). To develop a robust genetic variation inference, we implemented a two-step filtering approach consisting of a re-genotyping step followed by a back-genotyping approach. Re-genotyping determines the support of a specific variant in a given sample. For the back-genotyping, the inference procedure is performed against a simulated reference genome that includes the detected variants (S1 Fig). Variants that are not supported by back-genotyping were considered as undetermined in this sample and variants that were inferred to be undetermined in many samples are unreliable and discarded from the analysis (S2 Fig). Testing our approach using simulations showed that our variant calling approach retrieved 96.3% of simulated indels (S3 Fig).

Applying our approach to the CAO strains, we obtained 2369 variants; thereof 1435 variants were determined in every sample by back-genotyping (60.6% of all detected variants). A total of 356 (15.0%) variants were filtered as they were inferred as undetermined in many samples by the back-genotyping procedure (substitutions were excluded if they are undetermined in more than five samples and indels were excluded if they are undetermined in more than 20 samples, S2 Fig). Notably, our approach filtered out undetermined variants where the read coverage was not sufficient across all samples (S2F Fig) and variants inferred in genomic regions subject to putative rearrangements (S2G Fig). We further discarded 207 variants (8.7%) since their presence-absence patterns showed no variation (i.e., they are found in all or none of the samples, where they were determined).

After back-genotyping, we inferred in total 1806 high-quality variants, of which 467 (25.8%) were undetermined in some samples. High-quality variants comprise a majority of SNPs (1598, 88.5%) and 208 indels (11.5%), where the majority of inferred indels are short (≤50bp; Table 1, S4 Fig). We noted a peak in the distribution of insertion length around 1360bp that corresponds to 38 different insertions of the mobile element IS6110 (S4 Fig). IS6110 insertions are known to be found preferentially in certain genomic regions, i.e., insertional hotspots, where IS6110 insertions can confer a growth advantage [14]. The distribution of distances between all IS6110 insertions inferred in the CAO isolates revealed eight insertional hotspots, of which two have been previously described (S5 Fig) [14].

**Table 1:** Summary and genomic localization of detected variants in the CAO.

	SNPs	Insertions		Deletions		Total
		Short	Long	Short	Long	
**In gene**	1362 (85.23%)	48 (73.85%)	28 (63.64%)	65 (73.86%)	11 (100%)	**1514**
**Intergenic**	202 (12.64%)	13 (20%)	13 (29.54%)	19 (21.59%)	-	**247**
**In pseudogene**	34 (2.13%)	4 (6.15%)	3 (6.82%)	4 (4.55%)	-	**45**
**Total**	**1598**	**65**	**44**	**88**	**11**	**1806**
**Parsimony informative**	434 (27.2%)	18 (27.7%)	14 (31.8%)	16 (18.2%)	4 (36.4%)	**486**
**Compatible**	394 (90.8%)	11 (61.1%)	10 (71.4%)	13 (81.2%)	2 (50%)	**428**

Percentages were calculated based on the total number of variants in each variant class, except for the compatible variants, where the percentage was calculated based on the number of parsimony informative variants in each variant class. The majority of SNPs and indels are found in coding regions. However, the distribution of variants in protein-coding and intergenic regions differs significantly from the expectation by chance (Fisher’s exact test, p<0.01), where variants (247, 13.7%) are enriched in intergenic regions that span 7.8% of the genome.

In the Hamburg outbreak that includes 64 isolates sampled over 15 years, we inferred a total of 112 variants; thereof 101 (81.4%) were determined in every sample by back-genotyping and 11 were undetermined in some samples. The majority of the inferred variants are SNPs and most of the indels are short deletions (S6A Fig). Notably, two long insertions were inferred as alternative IS6110 insertion events. Note that the two data sets are not entirely relatable. Whereas the drug-sensitive Hamburg outbreak occurs in a restricted area in Germany and includes few cases, the drug-resistant CAO is a larger outbreak in multiple countries over 20 years where 353 samples are available. Accordingly, we detected a higher number of variants in the CAO compared to the Hamburg outbreak. Thus, we focused on the CAO, and the results will be compared to the Hamburg outbreak when feasible.

### Short indels contribute significantly to antibiotic resistance evolution

To infer the putative phenotypic impact of the inferred variants in coding regions, we examined their localization in genes of known function. For this purpose, we retrieved a list of ABR-conferring genes, i.e., genes where mutations were found to confer antibiotic resistance [29] (S2 Table). Additionally, we classified all MTB genes in two categories of essentiality, according to their requirement for growth *in vitro* (i.e., essential) or not (i.e., dispensable) [30]. In particular, we investigated the distribution of genetic variants in essential and ABR-conferring genes. Depletion of variants in specific gene categories indicates purifying selection acting on that category, whereas enrichment serves as an indication for positive selection.

**Fig 1:**
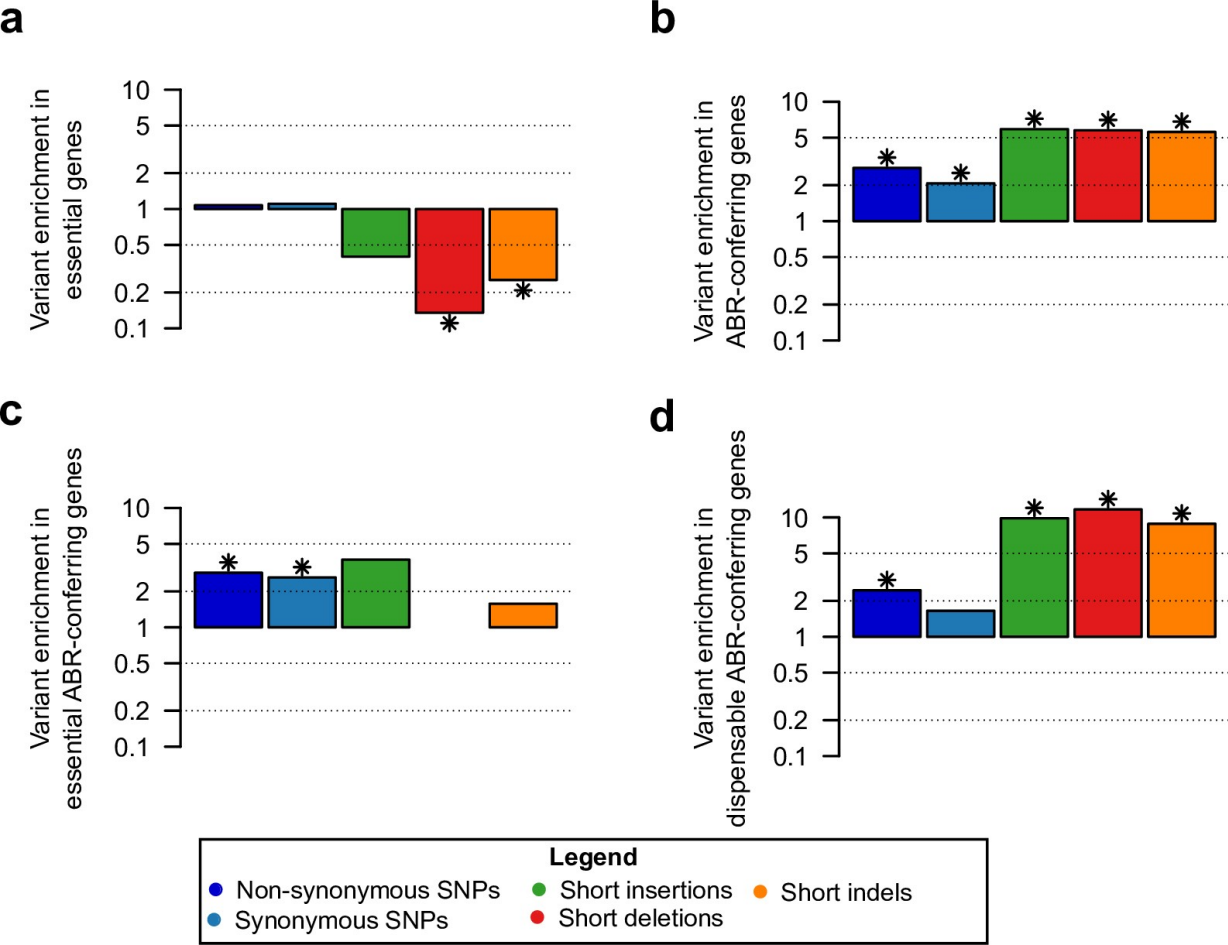
Enrichment analyses of variants in gene categories. (a) Essential, (b) ABR-conferring, (c) Essential and ABR-conferring, and (d) Dispensable and ABR-conferring. The variant enrichment was calculated as the ratio of the proportion of genes with variants in a gene category and the proportion of genes with variants outside the gene category. For example, three essential genes (0.6% of all essential genes) have short indels and the remaining short indels occur in 94 dispensable genes (3.3% of all dispensable genes), which results in a variant ratio of short indels in essential genes of 0.25, i.e., a fourfold depletion. Significant enrichment or depletion, marked by a star, was assessed using Fisher’s exact test (p-value < 0.05, corrected for false discovery rate (FDR), S3 Table).

First, we observed a fourfold depletion of short indel frequency in essential genes compared to dispensable genes; the distribution of SNPs, however, is not significantly different between essential and dispensable genes (Fig 1A). Furthermore, SNPs and short indels are enriched in ABR-conferring genes compared to the remaining genes (Fig 1B). When we classified the ABR-conferring genes into essential (27, 29.4% of ABR-conferring genes) and dispensable (65, 70.6% of ABR-conferring genes), we observed that SNPs are enriched both in ABR-conferring genes that are essential and in ABR-conferring genes that are dispensable. In contrast, short indels are significantly enriched in the ABR-conferring genes that are dispensable but not in the essential ABR-conferring genes (Fig 1C and 1D). In comparison, the drug-susceptible Hamburg outbreak did not show variants in ABR-conferring genes (S6 Fig).

The enrichment analyses highlight the selection pressures on SNPs and indels in the CAO. The depletion of short indels in essential genes provides evidence for the presence of strong purifying selection against indels in essential genes. In addition, the observed enrichment in ABR-conferring genes likely stems from the strong selection pressure on antibiotic resistance in the multi-drug resistant CAO. The significant enrichment of short indels in ABR-conferring genes that are dispensable shows that indels contribute to the evolution of antibiotic resistance in a highly resistant outbreak, potentially by frameshifts that disrupt the protein sequences.

### Insertions and deletions contribute to phylogenetic signal in MTB

**Fig 2:**
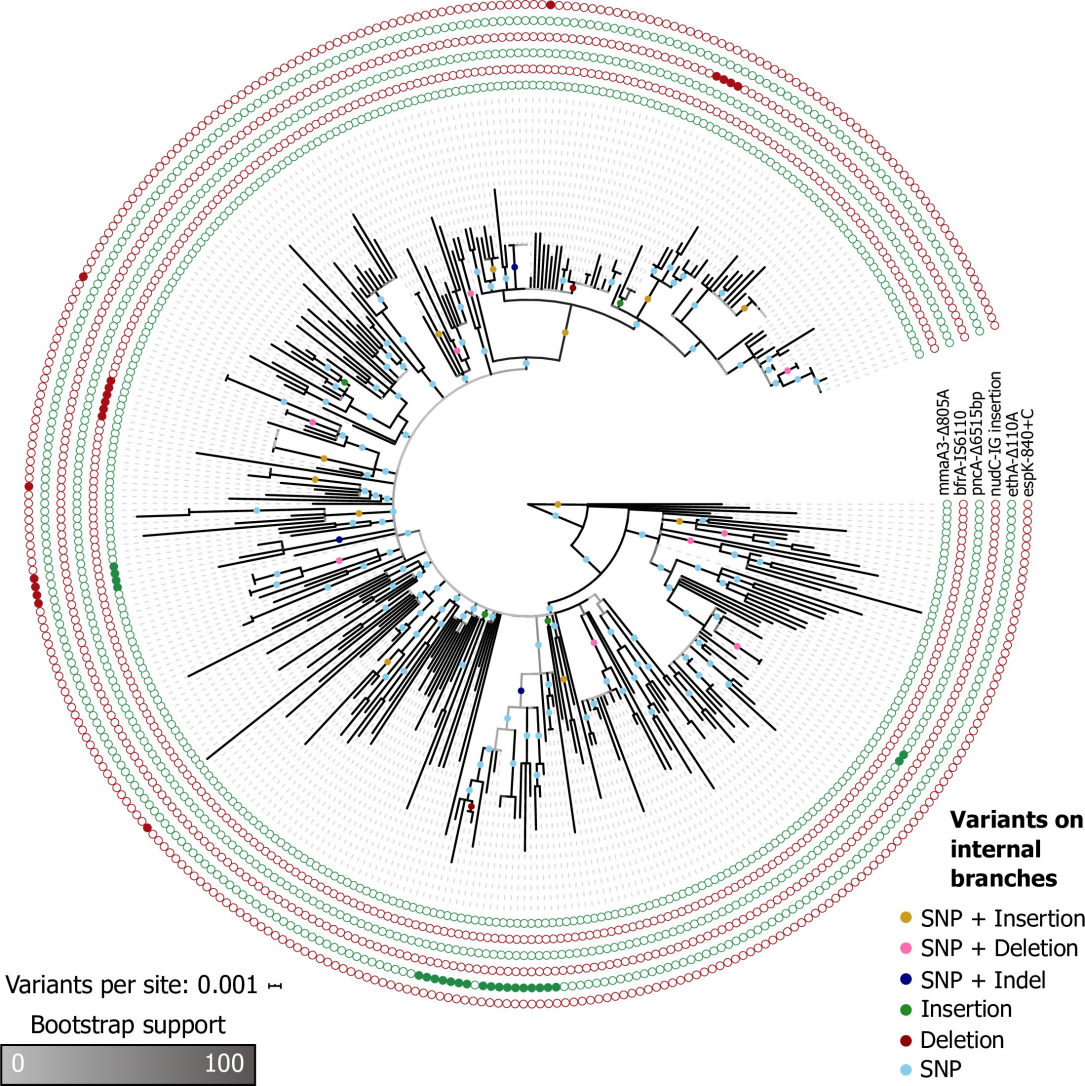
Phylogenetic tree of the CAO. 486 (26.9%) of the variants are parsimony informative, and 56 variants (3.1%) are incompatible with the tree topology (Table 1). The root position is the temporal root estimated by dating the phylogeny with LSD. Circles on branches represent variants that are compatible with the branch, i.e., they likely have emerged on that branch. We found that six branches in the tree have only short indels (four branches with insertions, two branches with deletions). The outer circles show example variants that are highlighted in the text. The tree with the tip labels is available in the supporting information (S7 Fig).

To study the transmission of indels in an outbreak, we next describe how the genetic variants are inherited in the CAO. To this end, we reconstructed the outbreak phylogeny from the presence-absence pattern of the variants in the strain genomes, where undetermined variants in a sample correspond to gapped positions. This analysis revealed that SNPs are the main contributors to the phylogenetic signal, where most of the parsimony informative SNPs are compatible with the phylogeny (Table 1). The inclusion of indels in the phylogenetic reconstruction increases the resolution of the tree topology at multiple places, where six internal branches are supported by a single short indel only (Fig 2). In the Hamburg outbreak phylogeny, two internal branches are supported by indels only (S6B Fig).

We also compared our approach to the standard SNP-based phylogenetic approach for the CAO data. The phylogeny reconstructed from all variants contains 157 resolved internal branches (i.e., length larger than zero), whereas the SNP phylogeny contains 149 resolved internal branches. Notably, 138 branches (92.6% of the branches in the SNP phylogeny) are found in both trees. Thus, our phylogenetic inference is consistent with the standard SNP-based phylogeny and additional branches are resolved. In conclusion, we find improved phylogenetic resolution for both the CAO and Hamburg outbreak, which demonstrates that indels can provide additional insights into possible transmission events.

### Substitutions and short indels in the CAO follow a molecular clock

**Fig 3:**
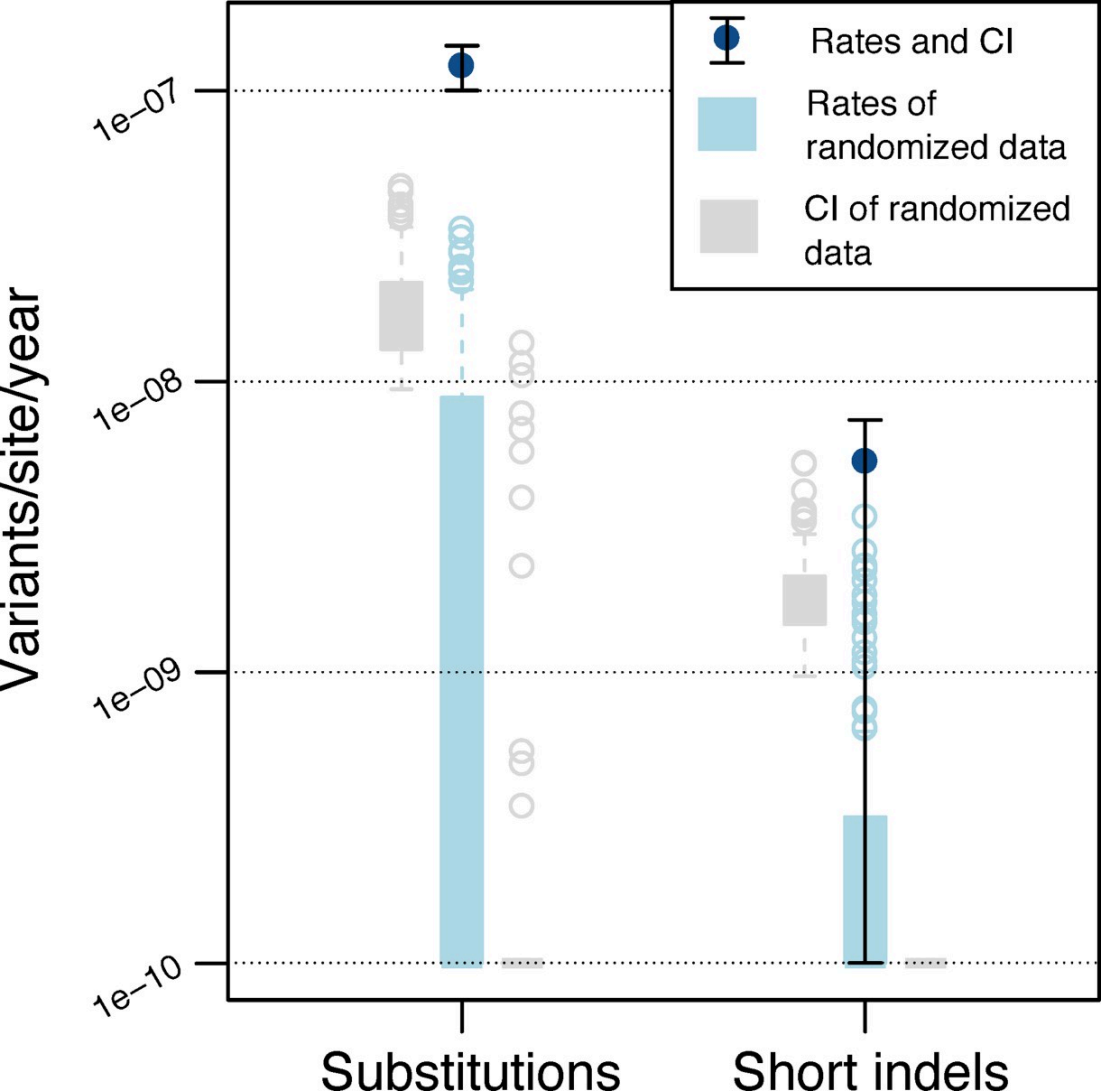
Evolutionary rates of substitutions and short indels and their associated 95% confidence intervals (CI) estimated with LSD for the CAO. Substitution rate was estimated as 1.23e-7 [1.00e-7–1.43e-7] substitutions/site/year and the short indel rate was estimated as 5.37e-9 [1e-10–7.37e-9] indels/site/year. The light colors show the distribution of rates for date-randomized data and their associated lower and upper confidence intervals; information for all randomized data sets are in the supporting information (S8 Fig). For substitutions, there is no overlap in rate and confidence interval between real data and date-randomized data, indicating the presence of temporal signal according to the stringent test. For short indels, the evolutionary rate inferred from the real data does not overlap with the rates of the date-randomized data. However, the confidence interval of the evolutionary rate inferred from the real data overlaps with the upper confidence intervals of the date-randomized data, indicating the presence of temporal signal for short indels according to the simple test. Hence, the temporal signal of short indels is weaker than that of substitutions. Due to the limited number of events, there is not sufficient temporal signal to estimate evolutionary rates for short insertions and deletions separately or for long indels.

To compare the dynamics of substitutions and indels in the outbreaks, we examined their evolutionary rates on the phylogeny. We followed the procedure for evolutionary rate estimation in MTB genomes developed in Menardo et al. [24]. To this end, we estimated evolutionary rates on the real data and on date-randomized data to determine the strength of the temporal signal according to three levels of significance that depend on the overlap between the estimates for the real data and for the date-randomized data (see methods). We found that substitutions in the CAO passed the stringent test for temporal signal, with an estimated rate of 1.23e-7 substitutions/site/year (Fig 3). For comparison, substitutions in the Hamburg outbreak passed the intermediate test for temporal signal, with an estimated rate of 7.51e-8 substitutions/site/year (S6C Fig). This rate is lower compared to the CAO rate; however, the confidence intervals of the estimates overlap. The substitution rate estimated here for the CAO is within the range of previously estimated lineage 2 rates [24]. Furthermore, there is a known difference in the rate of evolution between MTB lineage 2 and lineage 4 [31], which is consistent with our estimates for the lineage 2 CAO and the lineage 4 Hamburg outbreak.

We then estimated the evolutionary rate of indels in the CAO. Short indels are assumed to emerge by similar point mutation processes, in contrast to long indels that are due to segmental mutations (e.g., [32]). We thus considered short indels and long indels separately for the rate estimation, where only short indels have temporal signal (according to the simple test), with a rate of 5.37e-9 short indels/site/year (Fig 3). In comparison, indels in the Hamburg outbreak do not have temporal signal (S6D Fig). Hence, our analysis of variants in MTB lineage 2 strains revealed that substitutions and short indels follow a molecular clock, even though short indels exhibit weaker temporal signal than substitutions. Our results show that short indels evolve 23 times slower than substitutions. The difference between substitution and indel rates might be explained by the extremely efficient and redundant MTB repair mechanisms. Some error-prone repair mechanisms are known to introduce substitutions upon repairing DNA damage, for example, the DnaE2 pathway that is involved in trans-lesion synthesis [33]. Thus, MTB repair mechanisms might generate a mutational bias towards substitutions, leading to a stable genome with few structural variations over evolutionary time.

### Indels are subject to vertical inheritance and convergent evolution in the CAO

To infer the function of variants that are transmitted in the outbreak or that evolved multiple times independently, we explored the phylogenetic distribution and congruence of indel variants in the reconstructed CAO tree. Parsimony informative variants can be compatible with the phylogeny (termed compatible variants hereafter), i.e., they are inferred as vertically inherited and transmitted to multiple hosts. We thus hypothesize that the effect of compatible variants on MTB fitness is likely not highly deleterious; or they might even have an advantageous effect. In contrast, parsimony informative variants can be incompatible with the phylogeny (termed incompatible variants hereafter), i.e., they are convergent events where the same variant occurs independently in two or more disparate branches of the tree, indicating convergent evolution. Incompatible variants cannot solely be taken for evidence of positive selection; rather, they may indicate positive selection if they emerged in similar genetic backgrounds and have an identical phenotypic impact [34].

We found 394 compatible and 40 incompatible SNPs (9.2%) in the CAO. Incompatible SNPs are mainly found in ABR-conferring genes (21 out of 29 incompatible SNPs in coding regions, 72.4%, S5 Table). This observation is in agreement with previous results where convergent SNPs have been observed in MTB genes that confer antibiotic resistance and compensatory mechanisms of the antibiotic resistance fitness costs [17,22]. For indels, we found a total of 36 compatible and 16 incompatible indels (31.8%) in the CAO. Thus, incompatible indels are enriched among the parsimony informative indels in comparison to incompatible SNPs (p-value<0.01, Fisher’s exact test). The enrichment of incompatible indels can be traced back to short indels in homopolymer regions and also inference bias (S4 Table). We found that nine of the 16 incompatible indels are located in coding regions (56.2%; S4 Table). Thereof, eight indels are found in dispensable genes and two in ABR-conferring genes, where all incompatible indels in ABR-conferring genes are long deletions completely or partially deleting *pncA*. Furthermore, 30 of the 36 compatible indels are found in coding regions (83.3%; S4 Table), out of which 29 are in dispensable genes and two are in ABR-conferring genes (*mmaA3* and *ethA*; Fig 2).

We next considered genes in which multiple indels were inferred, i.e., genes affected by convergent evolution due to indels. We found 15 indels affecting four ABR-conferring genes; in three of these genes (*rpoB*, *tlyA*, *ethA*), additional SNPs were inferred in different samples, whereas no SNPs were inferred in *pncA* (S6 Table). In addition, twelve genes where multiple indels have been found do not confer antibiotic resistance according to our annotation (e.g., *espB* and four PE genes; S6 Table); these genes do not contain SNPs in any sample.

PE and PPE genes are repeat-containing coding sequences, whose products are secreted and are hypothesized to be important for MTB interaction with the host immune system [35]. An examination of all variants inferred in PE and PPE genes in the CAO strains revealed 17 short indels. Of these, only three (17.6%) cause a frameshift, which is much lower than the proportion of frameshift-causing indels in all coding sequences (77.8%). Furthermore, we found seven parsimony informative short indels, out of which six are compatible (five in-frame) and one is an incompatible in-frame deletion. Notably, the evolution of PE and PPE genes in the drug-sensitive Hamburg outbreak follows a similar pattern. There, we found seven deletions in PE and PPE genes, where six (85.7%) of the deletions are in-frame and four (57.1%) are compatible. The vertical inheritance and the enrichment of in-frame indels in PE and PPE genes indicate that these proteins are fast evolving in MTB outbreaks, further supporting the hypothesis that they are involved in host recognition [35].

Taken together, we found 52 parsimony informative indels in 34 different genes. Notably, only two of these genes (*ethA* and a nitronate monooxygenase) likely evolved under positive selection as inferred by the ratio of nonsynonymous to synonymous substitutions (dN/dS>1, S7 Table). We note that the inference of positive selection can only be performed for three of the 34 genes with parsimony informative indels due to the lack of SNPs in the remaining genes. We further discovered that four genes with parsimony informative indels (11.8%) are included in a set of 116 (3%) genes that were found to be under positive selection in a recent survey of dN/dS in MTB [36]. Thus, while indels can be found in genes that are under positive selection as calculated by the dN/dS ratio, they might also uncover additional genes involved in adaptation.

In the following, we study six example indels in detail that were selected among the parsimony informative indels to highlight possible convergent evolution, inherited antibiotic resistance, and co-occurrences of variants.

### An incompatible short insertion disrupts the type VII secretion system gene *espK*

**Fig 4:**
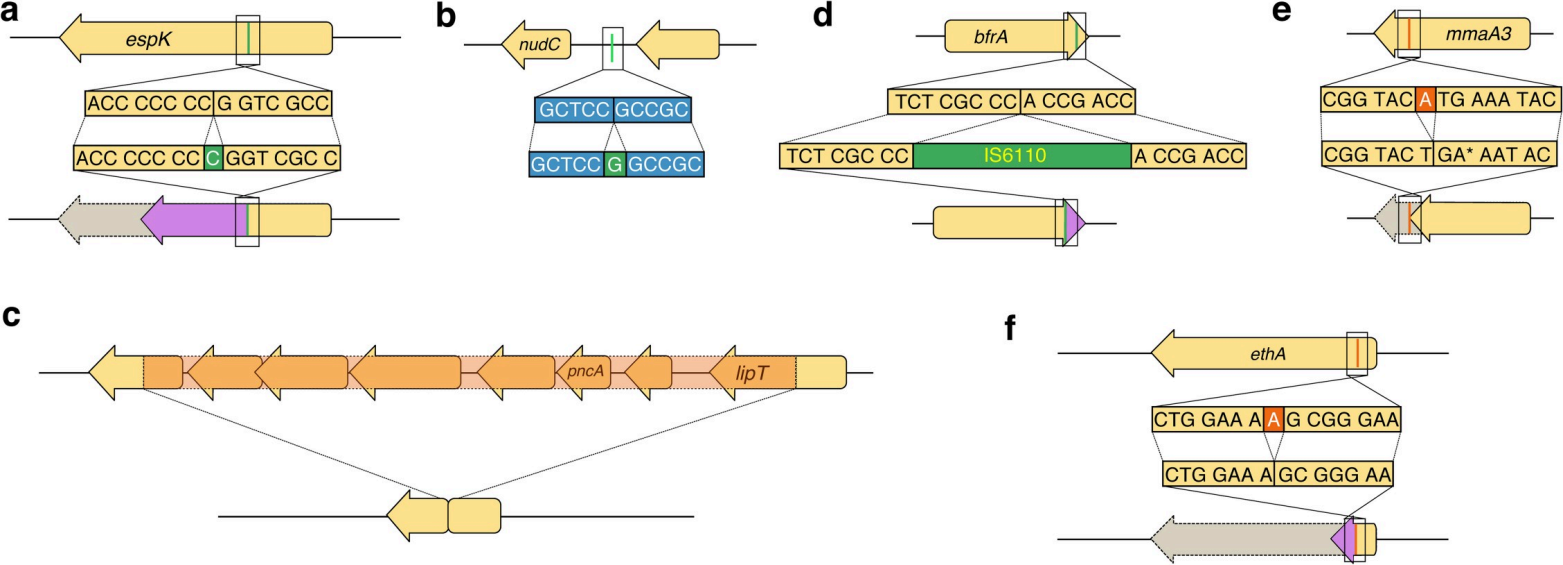
Examples of indels subject to vertical inheritance and convergent evolution. The orientation of the genes is relative to the reference genome 49–02. The ancestral (reference) sequence is shown at the top and the evolved sequence (including the variant) is shown at the bottom. See Fig 2 for phylogenetic locations. Gene names are displayed according to the Mycobrowser annotations (https://mycobrowser.epfl.ch/, last accessed 28 November 2019), when available. Following annotations are shown as (Locus tag in 49–02, homolog in H37Rv). (a) Single base-pair insertion in the *espK* gene (MT49_RS20440, Rv3879c). This insertion occurs at position 840 of the coding sequence (37.7%), resulting in a truncated protein of 465 amino acids (62.7% of the wild type length). (b) Intergenic single base-pair insertion 39bp upstream of the *nudC* gene (MT49_RS16870, Rv3199c). We could not detect the promoter sequence in this intergenic region using BPROM [40]. (c) 6515 base-pair deletion removing the *pncA* gene (MT49_RS10715, Rv2043c) and five of its neighboring genes. The left breakpoint is located at position 529 in the gene *ugpC* (MT49_RS10690, Rv2038c). The right breakpoint is located at position 453 in a gene encoding for a carboxylesterase/lipase family protein (MT49_RS10725, Rv2045c). Four of the deleted neighboring genes encode for ABC transporters (S4 Table). Two variants co-occur with this long deletion (Table 2). (d) IS6110 integration close to the 3’ end of the *bfrA* gene (MT49_RS09760, Rv1876). The integration occurs at position 471 of the coding sequence (98.1% of total CDS length), resulting in a protein of 162 amino acids (2 amino acids longer than the wild-type), with the last two amino acids of the wild type different in the mutated protein. We found eight co-occurring variants on the same internal branch (Table 2). (e) Single base-pair deletion close to the 5’ end of *mmaA3* (MT49_RS03370, Rv0643c). The deletion removes the residue at position 805 of the coding sequence, resulting in a stop codon where the mutated protein (269 amino acids) corresponds to 91.5% of the wild-type protein. Six variants co-occur on the same internal branch (Table 2). (f) Single base-pair deletion in the beginning of the *ethA* gene (MT49_RS20315, Rv3854c). This deletion occurs at position 110 of the coding sequence (7.5% of the CDS length), which results in a frameshift where the resulting protein is truncated with a length of 62 amino acids long (12.6% of the wild type length). This deletion co-occurs with two variants (Table 2).

We observed a 1bp insertion in the *espK* gene in eight samples including four related samples and four unrelated samples (Fig 2). Hence this insertion likely emerged five times independently, indicating convergent evolution. The variant emerged in a homopolymer region of seven cytosines (Fig 4A), resulting in a frameshift and a premature stop codon. The *espK* gene is located in the ESX-1 locus, a type VII secretion system. The locus additionally comprises PE and PPE genes, encoding for proteins that are exported or found in the cell membrane [37]. EspK is thought to act as a chaperone of the neighboring *espB* gene [38], and is found dispensable for growth *in vitro*. EspB acts as a repressor of the host immune response, thereby increasing MTB survival [39]. Notably, it was shown that inhibition of *espK* and *espB* results in reduced virulence in comparison to the wild-type [39]. The 1bp insertion likely renders EspK nonfunctional and hence has a direct effect on EspB function as well. Previous studies observed convergent substitutions in another type VII secretion system gene (*esxW* in ESX-5) that increased MTB transmissibility [23]. Hence, the 1bp indel in *espK* might influence MTB transmissibility as well.

### An intergenic short insertion is located upstream of the ABR-conferring *nudC*

A compatible 1bp insertion located in an intergenic region 39bp upstream of *nudC* was observed in four related samples (Fig 2, Fig 4B). NudC is an NAD(+) diphosphatase, where antibiotic resistance to isoniazid and ethionamide was observed when overexpressing *nudC* [41]. Of note, the *nudC* gene in the outbreak reference genome 49–02 has a 239P->239R polymorphism compared to the reference H37Rv. Substitutions at this position have been found to disrupt the NudC dimer formation; thus, it is expected to affect the NudC catalytic activity [41]. The observed indel is compatible; hence, we hypothesize that the short insertion is advantageous to MTB, for example by altering the *nudC* expression level.

### A long deletion completely removes the ABR-conferring *pncA* and neighboring genes

A compatible 6515bp deletion of *pncA* with five neighboring genes was observed in two related samples (Fig 2) and is co-occurring with two substitutions (Table 2). The long deletion furthermore disrupts two additional neighboring genes and results in a chimeric coding sequence (Fig 4C). PncA activates pyrazinamide, a first-line antibiotic of the category “prodrug”, i.e., a compound that needs to be activated to exhibit toxicity. The disruption of *pncA* renders pyrazinamide inactive and thus results in antibiotic resistance; hence, indels in *pncA* have been previously observed to confer resistance to pyrazinamide [42]. In our data, in addition to the multi-gene deletion, we observed a complete *pncA* deletion and nine disruptive indels across the tree (four short insertions, one long insertion, and four long deletions). Thus, *pncA* has the highest frequency of convergent indels in the CAO, which supports the reports that *pncA* exhibits high levels of homoplasy [17].

**Table 2:** Variants that co-occur with the example indels (Fig 2).

Co-occurring variant	Locus tag	H37Rv homolog	Gene name	Product	Notes
6515bp deletion of *pncA*					
iSNP	MT49_RS03955	Rv0755c	PPE12	PPE family protein PPE12	
sSNP	MT49_RS13630	Rv2582	*ppiB*	peptidyl-prolyl cis-trans isomerase	
IS6110 insertion into *bfrA*					
nsSNP	MT49_RS03540	Rv0676c	*mmpL5*	siderophore RND transporter MmpL5	ABR-conferring
sSNP	MT49_RS04920	Rv0935	*pstC1*	phosphate ABC transporter permease	
nsSNP	MT49_RS08525	Rv1625c	*cya*	adenylate cyclase	
nsSNP	MT49_RS09530	Rv1830		MerR family transcriptional regulator	
sSNP	MT49_RS09595	Rv1843c	*guaB1*	GuaB1 family IMP dehydrogenase-related protein	
nsSNP	MT49_RS12315	Rv2337c		hypothetical protein	
nsSNP	MT49_RS20315	Rv3854c	*ethA*	FAD-containing monooxygenase EthA	ABR-conferring
1bp deletion in *mmaA3*					
sSNP	MT49_RS03380	Rv0645c	*mmaA1*	mycolic acid methyltransferase MmaA1	Involved in membrane biogenesis
iSNP	MT49_RS08155	Rv1535		hypothetical protein	
IS6110 insertion	MT49_RS16445	Rv3126c		hypothetical protein	
nsSNP	MT49_RS17845	Rv3383c	*idsB*	polyprenyl synthetase family protein	Involved in membrane biogenesis
nsSNP	MT49_RS19685	Rv3740c		wax ester/triacylglycerol synthase family O-acyltransferase	Involved in membrane biogenesis
nsSNP	MT49_RS20030	Rv3800c	*pks13*	acyltransferase domain-containing protein	Involved in membrane biogenesis
1bp deletion in *ethA*					
nsSNP	MT49_RS03490	Rv0667	*rpoB*	DNA-directed RNA polymerase subunit beta	Essential and ABR-conferring
Intergenic IS6110 insertion	MT49_RS09400	Rv1804c		hypothetical protein	Predicted secreted protein

Gene names and additional information are displayed according to the Mycobrowser annotations (https://mycobrowser.epfl.ch/, last accessed 28 November 2019), when available. Locus tags and products are taken from the annotation of the 49–02 reference genome. In case of intergenic variants, we show the information for the gene downstream of the variant. Notes show additional information related to protein functions. iSNP–intergenic SNP, sSNP–synonymous SNP, nsSNP–nonsynonymous SNP.

### An IS6110 insertion elongates a bacterioferritin gene

A compatible insertion of an IS6110 element was identified at the 3’ end of *bfrA* in six related samples (Fig 2; Fig 4D). The deleterious effect of indels at the 3’ end of genes is considered minimal due to the indel location at the end of the open reading frame, thus maintaining the majority of the coding sequence [43]. Indeed, the IS6110 element insertion yields a protein sequence that is two amino acids longer than the wildtype, where 158 amino acids (98.8%) of the wild type protein are retained (Fig 4D). BfrA is an essential component for iron storage and distribution depending on iron availability [44] and it is classified as dispensable *in vitro*. We observed eight additional SNPs that co-occur with the IS6110 insertion in the same six samples (Table 2). Two of the SNPs are non-synonymous substitutions in ABR-conferring genes: *mmpL5* and *ethA*. MmpL5 is annotated as a siderophore transporter and variants can confer bedaquiline resistance; EthA variants can confer resistance to ethionamide [3]. Of the remaining co-occurring SNPs, one non-synonymous substitution is observed in a gene encoding for a protein related to iron export and one non-synonymous substitution is found in a MerR transporter family that plays a role in responding to environmental stresses, such as oxidative stress, heavy metals or antibiotics [45] (Table 2). Thus, the IS1660 insertion in *bfrA* might hitchhike with the co-occurring substitutions in *mmpL5* and *ethA* or it might even be a compensatory variant for the substitutions that confer antibiotic resistance and may be additionally related to iron transport and storage.

### A short deletion shortens the ABR-conferring gene *mmaA3*

A compatible deletion of one base pair was observed in the gene *mmaA3* in four related samples (Fig 2). The deletion results in a frameshift and a premature stop codon yielding a truncated protein sequence (Fig 4E). The protein MmaA3 acts along the synthesis pathway of mycolic acids, which are essential components of the bacterial membrane [46]. The gene is classified as ABR-conferring and as dispensable *in vitro*. In addition, we observed five SNPs and one IS6110 insertion that co-occur with the 1bp deletion in the same four samples (Table 2). Three of the five SNPs are non-synonymous substitutions in genes that encode proteins involved in membrane biogenesis (Table 2). Our results thus revealed several substitutions and indels, which emerged and were vertically inherited together, and which likely have an effect on the function of membrane biosynthesis genes.

### A short deletion disrupts the ABR-conferring *ethA*

A compatible 1bp deletion was observed in the *ethA* gene in 17 samples with an additional undetermined sample (Fig 2). This deletion results in a frameshift leading to a truncated protein (Fig 4F). EthA, an FAD-containing monooxygenase, is involved in the activation of ethionamide, a second-line antibiotic prodrug. The downregulation of *ethA* has been demonstrated to generate an ethionamide resistance phenotype [47]. We observed two additional co-occurring variants in the same samples, where one is a non-synonymous substitution in *rpoB* (Table 2). We hypothesize that the disruption of EthA confers a strong selective advantage by mediating resistance to ethionamide. RpoB is known to confer resistance to rifampicin upon mutations in the rifampicin resistance determining region, whereas mutations outside the rifampicin resistance determining region might compensate the cost of the resistance [10]. Here we observed a substitution outside the rifampicin resistance determining region and thus hypothesize that it might be involved in compensation, resulting in the vertical inheritance of the variants. Alternatively, both variants could confer antibiotic resistance. Interestingly, we found 20 additional variants in *ethA* throughout the tree, of which 17 are non-synonymous SNPs and three are single base pair deletions that cause a frameshift. The presence of SNPs that are exclusively non-synonymous shows that this gene is under strong positive selection (S7 Table). Our examples demonstrate that indels contribute to the evolution and diversification of MTB CAO strains, by affecting essential metabolic pathways and antibiotic resistance with potential pathobiological consequences. Furthermore, we found that compatible indels often co-occur with substitutions that affect related functions or pathways (Table 2); thus, indels might compensate the effects of substitutions or *vice versa*. In addition, the significant enrichment of short indels in ABR-conferring genes that are dispensable shows that indels contribute significantly to ABR evolution in the multi-drug resistant CAO, likely by frameshifts that disrupt the protein sequences. Our study demonstrates that, even if rare, including indels in outbreak genome analyses supplies crucial evidence for outbreak reconstruction and for the profiling of antibiotic resistant strains.

## Discussion

The contribution of indels to genome evolution is often understudied, mainly due to difficulties in reliable indel detection methodology. Our approach allows to infer high-quality indels by estimating the level of indeterminacy, which was used to identify unreliable genetic variants. MTB outbreak reconstructions so far mainly relied on the estimation of phylogenies based on SNPs. Here we find that six branches of the CAO tree and two branches of the Hamburg outbreak tree were reconstructed solely by short indels. Thus, we show that indels can be employed to increase the resolution of MTB strain comparisons in genomic epidemiology approaches, e.g., for outbreak investigations.

Furthermore, the accurate detection of indels in the CAO revealed an indel evolutionary rate that is lower than the substitution rate. It is known that the MTB mutation rate, that is, the rate at which mutations arise in the genome, is in the range of bacterial mutation rates (between 1.4e-10 for *Thermus thermophilus* and 4e-9 for *Buchnera aphidocola*; 1.9e-10 mutations/bp/generation for *M*. *tuberculosis* [48]). However, MTB strain evolution is characterized by a long generation time (i.e., cellular division cycles) [1] and strong purifying selection that eliminates most genetic variants from the population, with only few mutations being fixed [49]. Both of these processes contribute to a low substitution rate in MTB strains compared to strains of other bacterial species [6]. A previous comparison between evolutionary rates of mutations and indels in multiple bacterial species showed a 2.8 to 9.7-fold decrease of indel rates compared to mutation rates [50]. Notably, the comparison in the latter study is based on *de novo* rates, i.e., variants that arise in a bacterial individual per generation, which aim to include all variants before selection. In contrast, outbreak analyses include only the variants that are observed after the effect of selection. Hence, the 23-fold decrease of the MTB indel rate compared to the substitution rate can reflect both a lower *de novo* indel rate, as observed for other bacterial species, and a stronger effect of purifying selection on indels. The latter is expected since indels incur a higher fitness cost than substitutions as they often disrupt genes and render a truncated gene product [50].

Finally, indels are an important factor in the evolution of antibiotic resistance in MTB, where compatible and incompatible indels represent putative targets for positive selection. Notably, MTB is evolving strictly vertically without the contribution of recombination. The advantage of sex and recombination is widely discussed (e.g., [51]). On the one hand, recombination is beneficial by combining advantageous alleles from different genotypes in the population; whereas in the absence of recombination, advantageous alleles are linked to the genetic background where they arise (the Hill-Robertson effect). This genetic linkage might result in the fixation of neutral or slightly deleterious alleles by genetic hitchhiking with advantageous alleles. In addition, clonal interference between beneficial alleles in different genetic backgrounds slows down adaptation (the Fisher-Muller effect). On the other hand, in the presence of positive epistasis, i.e., when the double mutant has a higher fitness than expected from the individual alleles, recombination can lead to a decrease in fitness by breaking up advantageous allele combinations (resulting in recombination load). It has been observed that the magnitude of recombination impacts the genetic architecture of a species, where positive epistasis evolved in a bacteriophage model system under low recombination but not under high recombination [52]. In addition, an artificial gene network model has been used to demonstrate that positive epistasis evolves in asexual populations whereas negative epistasis can evolve in sexual populations [53]. It is thus expected that the asexual lifestyle of MTB results in a genetic architecture with widespread positive epistasis.

Indeed, epistatic interactions between genetic variants are widespread in MTB, where the emergence of ABR-conferring mutations is often accompanied by compensatory mutations to alleviate the fitness cost of antibiotic resistance [3]. The fixation of compensatory variants might even be favored over the reversal of antibiotic resistance in the absence of ongoing antibiotic treatment, because compensatory mutations might appear with a higher rate compared to the very specific target of a reversal mutation [54]. After the compensatory mutation increased in frequency, a newly appearing reversal mutation will not establish in the population due to clonal interference. Subsequently, transmission bottlenecks might contribute to the fixation of the compensatory mutation, after which the reversal mutation has only a low or no selective advantage precluding its establishment in the population [54]. Thus, in the MTB genetic architecture with widespread positive epistasis, compensatory variants might have a higher likelihood of being fixed compared to reversal mutations, even when the combination of resistance and compensatory mutation has a lower fitness than the reversal mutation. This evolved genetic architecture thus supports the fixation of compensatory mutations instead of reversal mutations.

Here we highlight that inherited indels are found to co-occur with substitutions. Although some of the co-occurring variants might be explained by genetic hitchhiking, the presence of co-occurring indels and substitutions in related gene functions or pathways supports that these variants interact epistatically. Since the contribution of horizontal mechanisms to MTB evolution is thought to be negligible, we thus conclude that MTB evolved a genetic architecture dominated by genetic linkage and positive epistasis, where epistasis between substitutions and indels contributes to the establishment of indels in the population.

Taken together, our results demonstrate the interplay of substitutions and indels in the evolution of biological functions that are essential for MTB infection and antibiotic resistance. We identified short and long indels that improve the resolution of outbreak phylogenies and that are crucial for the prediction of drug resistance in MTB strains. Especially for new hallmark drugs to treat multi-drug resistant MTB, such as bedaquiline and clofazimine, indels play a major role in collateral resistance towards both drugs [55,56]. Thus, increasing knowledge on interactions between all variant types is paramount for our understanding of the fundamental evolutionary principles that govern the spread of antibiotic resistance and the associated compensatory mechanisms in MTB.

## Methods

### Sample collection and variant calling

We analyzed 353 multi-drug resistant MTB strains sampled longitudinally from the Central Asian Outbreak (MTB lineage 2, first referenced in [27]). The closely related and fully drug-susceptible strain 49–02 (RefSeq: NZ_HG813240.1 version 11-MAR-2017) served as the reference genome for variant calling. In addition, we performed the analysis for an outbreak of 64 fully drug-susceptible isolates in the Hamburg region (S1B Table, MTB lineage 4, first referenced in [28]). Variants were inferred on the complete reference genome 7199–99 (RefSeq: NC_020089.1 version 19-MAY-2017).

We first trimmed the reads using trimmomatic v. 0.36 [57], with parameters SLIDINGWINDOW:4:15 MINLEN:36 LEADING:3 TRAILING:3. We mapped the trimmed reads to the outbreak reference genome using BWA MEM v0.7.16 [58], realigned around indels with GATK v3.8-0-ge9d806836 [59], and marked duplicates with PICARD v2.13.2. The median coverage ranges from 41 to 255. To detect SNPs and indels, we combined seven variant calling tools: GATK v3.8-0-ge9d806836 [59], FreeBayes v1.1.0-50-g61527c5 [60], Delly v0.7.7 [61], Pindel v0.2.5b9 [62], SvABA FH Version 134 [63], Scalpel v0.5.3 [64], and MindTheGap v2.0.2 [65]. Tools were run and variants filtered according to tool-specific quality scores (S8 Table) and we retained variants with a frequency over 75%.

It is known that many indels occur in genomic regions of high GC content or that contain tandem repeats [43]. The alignment of reads to these regions is therefore more difficult and determining indels is more complicated than determining SNPs. Notably, in the case of MTB, this has led to the systematic exclusion of variants in PE and PPE genes [21]. Here we implement two filtering steps, re-genotyping and back-genotyping, to obtain high-quality genomic variants.

### Re-genotyping

Since variants can be missed by variant calling, we performed re-genotyping to ascertain presences and absences of each variant in every sample. All samples are re-genotyped for all variants detected in any sample. We used GATK to re-genotype SNPs and short indels and svtyper [66] to re-genotype long deletions (S8 Table). Recommended hard filtering was applied to the variants genotyped with GATK and all re-genotyped variants having a frequency over 75% were retained. Since no tool, to our knowledge, can be used to genotype long insertions, we used the breakpoints identified by MindTheGap as evidence of presence. As re-genotyping was performed with a single tool per variant category, it standardizes the variant detection, allowing for a comparable assessment of variants from the same category. Similar indel variants are grouped together and considered as identical in downstream analyses if they co-cover each other by at least 95%.

### Back-genotyping

To quantify if variants can be determined by variant calling, we implemented an additional layer of filtering for ambiguous signal. The idea behind the back-genotyping is to identify the “inverse signal” of a variant to confirm the presence or absence of a variant (S1 Fig). The approach consists of (i) generating multiple modified genomes of the outbreak reference that contain the detected variants, (ii) mapping the reads of each sample to each of the modified genomes, and (iii) genotyping the variant positions as for re-genotyping. For SNPs, we genotyped the variants as they were introduced. For insertions, we genotyped the corresponding deletions; for deletions, we genotyped the corresponding insertions. To generate variant genomes, we included either SNPs at least 200 bp apart or indels at least 5000 bp apart (95 genomes for 2369 variants in the CAO, 12 genomes for 130 variants in the Hamburg outbreak).

Variants in genomic regions that are difficult to align are expected to be undetermined in many samples, i.e., they show inconsistent results for genotyping and back-genotyping. Therefore, we filtered SNPs if they are undetermined in more than five samples, and the remaining variants if they are undetermined in more than 20 samples. These thresholds have been established in order to limit the number of incompatible variants in the final set of variants, of which some were manually verified in IGV [67] (S2 Fig).

### Simulations

To assess the performance of variant calling and back-genotyping, we performed a simulation analysis. For this purpose, we introduced variants into the Hamburg outbreak reference (RefSeq: NC_020089.1). To assess SNP calling, the genome was modified 50 times, each containing 50 SNPs (introduced randomly at least 2,500bp apart). To assess indel calling, the genome was modified 620 times, each containing either one insertion or one deletion (randomly introduced), where ten replicates per indel length, ranging from 1bp to 10,000bp, have been carried out (S3 Fig). Next, we simulated reads on each of the modified genomes using the ART package v.2.5.8 [68]. We first generated a reads profile using art_illumina_profiler and decided, for conservative purposes, to take the profile of the shortest read length in both our data sets (i.e., reads of length 100bp from the Hamburg outbreak, S8 Table). Then, we generated the read data sets with art_illumina with a median coverage of 120, insert size 300 and standard deviation of 120 (S8 Table). The reads were then mapped back to the outbreak reference and variants were called and re-genotyped. Next, we back-genotyped the variants found for a data set. To this end, we mapped the simulated reads to the outbreak reference or, in the case of a false positive, to a modified genome that includes the inverse of the variant.

### Functionality assignments & enrichment tests

Since functions of MTB genes are determined based on experiments in H37Rv, we first retrieved homologs between the outbreak references and H37Rv (NC_000962.3). For this, we performed a blastp [69] between the protein sequences of each reference and H37Rv and retrieved the significant hits (e-value < 1e-10). After computing the global identity between significant hits with the needle algorithm [70,71], we considered proteins as homologs if they shared a global identity higher than 30%. We then assigned the six essentiality categories described in [30] to the homologous proteins in the outbreak references and grouped the gene categories into two main ones: essential genes, i.e. genes that are required for growth *in vitro*, and dispensable genes, i.e. genes that are not required for growth *in vitro*. The latter category includes genes annotated as non-essential, conferring growth advantage, conferring growth defect, uncertain and containing an essential domain with the addition of genes without homolog in H37Rv.

A list of genes that were found to confer drug-resistance upon mutations in H37Rv are additionally used for annotation ([29], S2 Table). We identified the homologs in the outbreak reference and assigned the ABR-conferring or non-ABR-conferring categories accordingly.

### Phylogeny inference and evolutionary rate estimation

The back-genotyping allowed us to generate a presence-absence matrix, which encodes the presences and absences of variants in all samples of our dataset. Variants that were undetermined in a sample are represented as gaps. We estimated the phylogeny based on the presence-absence patterns of the final variants with iqtree v1.6.1 [72], using the GTR2+FO binary model, and the ultrafast bootstrap. We displayed the tree using iTol v4.3.3 [73]. The position of the root was estimated by the least-squares dating method implemented in LSD v0.3beta [74]. Parsimony informative variants contain information on the phylogenetic relationships and are defined as being absent and present at least two times each in the presence-absence pattern.

We used LSD v0.3beta [74] to estimate evolutionary rates. For each class of variants, we extracted their presence-absence sub-matrix, estimated the branch lengths on the pre-estimated phylogeny (iqtree option–pre). To assess temporal signal, we randomized the dates 100 times and repeated the estimation [75,76]. The estimated evolutionary rates are considered significant with three levels of significance following Menardo et al. (2019) [24]. The stringent test assigns significance if the rate confidence interval of the real data does not overlap with any of the confidence intervals of the date-randomized data sets. The intermediate test assigns significance if the rate of the real data does not overlap with any of the confidence intervals of the date-randomized data sets. Finally, the simple test assigns significance if the rate of the real data does not overlap with the rates of the date-randomized data sets [24]. The hard limit of 1e-10 is imposed by LSD.

## Supporting information

S1 Fig Schematic summary of the back-genotyping. The example shows the calling and back-genotyping of a small deletion. (A)-(B) We identify a short deletion in one sample (blue). We include this deletion in the modified genome and map all the samples, and we genotype variants. (C)-(D) We either detect an insertion (yellow sample), if the base pairs that are removed in the modified genome are present in the reads, or nothing if the deleted portion is not present in the reads (grey sample). (E) The decision table summarizes the decision on the variants for each sample. If a variant is detected in the first phase and the inverse variant is not detected in the second phase, the variant is considered present. Alternatively, if the variant is not detected in the first phase but the inverse variant is detected in the second phase, the variant is considered absent. For the remaining cases, the variant is considered undetermined. These decisions are summarized in a presence-absence matrix, where the variants are denoted as present (1), absent (0), or undetermined (gap, “-“).
(TIF)

S2 Fig Undetermined variants. (A) Parsimony scores of SNPs having at most five gaps (i.e., SNPs that are undetermined in at most five samples), (B) Parsimony scores of SNPs having between six and 20 gaps. (C) Parsimony scores of SNPs having more than 20 gaps, (D) Parsimony scores of indels having at most 20 gaps, (E) Parsimony scores of indels having more than 20 gaps. The parsimony scores of SNPs between six and 20 gaps are high (up to 36), therefore we excluded SNPs having more than five gaps. Indels exhibit rather low parsimony scores (one to five) for variants exhibiting at most 20 gaps. Variants having more than 20 gaps have higher parsimony scores (up to 20), therefore the threshold has been set to 20 gaps for including indels. (F) Example of a deletion of 9bp that is undetermined in some samples displayed with IGV. This deletion was detected as present in seven samples (here 7542–15) and undetermined in 118 samples (here 2196–00, 10037–02 and 10540–05), hence it was filtered out according to our criteria. This example illustrates the importance of back-genotyping for real sequencing data in genomic regions that are difficult to align. Here, the coverage in the samples where the deletion was not detected is nearly zero, thereby not allowing for a confident variant inference. (G) Example of a deletion of 438bp that is undetermined in some samples displayed with IGV. This deletion was detected as present in 133 samples (here 7051–15) absent in two samples (here 3413–10) and undetermined in 218 samples (here 9545–15 and 3523–15). In samples where the deletion is undetermined, while we see a decrease of coverage potentially associated with a deletion, the reads tend to show rearrangements (i.e., displayed as different read colors in IGV). Assigning absences would have potentially led to a misleading phylogenetic reconstruction since we cannot ascertain the presence or absence of these variants in the low-covered samples or samples with putative rearrangements.
(TIF)

S3 Fig Number of true positive variants in simulations. Ten replicates have been carried out for each setting. (A) Short insertions, (B) short deletions, (C) long insertions, (D) long deletions, (E) table of variants found by each step of the variant calling. The combination of the variants detected by each variant calling tool allowed to retrieve a total of 3,104 variants (99.5%). In addition, we found 157 false positives (17 SNPs and 140 indels up to 77bp) with GATK and Freebayes when calling long indels (>50bp). Since these tools are not designed to find such long variants, false positives could occur due to misalignment of the reads in missing or added portions of the genome. After re-genotyping, there are 3,078 true variants (600 indels, 96.8% of all indels) and 144 false positives. All these variants were also back-genotyped. The high sensitivity of the approach in simulations reflects, in part, that the variants were included in the same genomic backbone that was used as a reference genome.
(TIF)

S4 Fig Cumulative length distribution of 208 indels. 65 of 44 insertions are short (59.6%) and 88 of 99 deletions are short (88.9%).
(TIF)

S5 Fig Cumulative distribution of the distance between neighboring IS6110 elements. The grey dotted line shows the expected distance between neighboring IS if they were uniformly distributed along the genome (116,115 bp). Here, we defined an IS6110 insertional hotspot as at least two insertions, distant of at most 5,000 base pairs (red line), in two different samples. The distance cutoff was chosen since it includes ten of 38 neighboring pairs (26.3%). In our data, we could classify 17 of the 38 IS insertions (44.7%) into eight hotspots. Two of the hotspots have already been reported and they correspond to insertions into the DnaA-DnaE intergenic region (region 3013bp– 3237bp in the genome) and the phospholipase C region (2,623,208bp– 2,625,716bp). The six remaining hotspots are located in genomic regions consisting mainly of hypothetical proteins (regions in the genomes are: 2,030,702bp– 2,031,324bp, 2,551,089bp– 2,554,757bp, 2,574,702bp– 2,575,128bp, 2,610,662bp– 2,610,752bp, 3,481,063bp– 3,482,894bp and 3,545,241bp– 3,545,781bp).
(TIF)

S6 Fig Genomic localization, phylogenetic tree, evolutionary rates and enrichment analysis for 64 strains sampled from the “Hamburg” outbreak. The Hamburg outbreak is a fully-sensitive MTB strain of lineage 4 sampled in the Hamburg and Schleswig-Holstein region between 1998 and 2013 (S1B Table; Roetzer et al., 2013 [28]). (A) Summary and genomic localization of detected variants. Percentages were calculated based on the total number of variants in each variant class, except for the compatible variants, where the percentage was calculated based on the number of parsimony informative variants in each variant class. The two long insertions correspond to IS6110 insertions, inferred as two alternative insertions in a pseudogene annotated as a Fic protein. No variants were found in ABR-conferring genes and the enrichment analysis on the essential category did not show any significant results. (B) Phylogenetic tree of the Hamburg outbreak, inferred from the presence-absence patterns of 112 detected variants and rooted by the temporal root estimated with LSD. Our approach found that all variants but one deletion are compatible with the phylogeny. Notably, two branches are refined by single indels. One is an IS6110 insertion that groups two samples, the second is a 45bp deletion in a PE gene that groups nine samples. (C) Substitution rate and 95% confidence intervals (CI) estimated with LSD. Substitutions have temporal signal according with the intermediate test for temporal signal, with an estimated substitution rate of 7.51e-8 [2.85e-8–11.0e-8] substitutions/site/year, where the confidence interval (CI) includes the previous estimate of 1e-7 substitutions/site/year (Roetzer et al., 2013 [28]). (D) Short indel rate and 95% confidence intervals. The overlap between the rates of the date-randomized data shows that there is not sufficient temporal signal to estimate evolutionary rates for indels. The absence of temporal signal might be traced back to the low number of indels in this data set.
(TIF)

S7 Fig Phylogenetic tree of the CAO with tip labels. The root position is the temporal root estimated by dating the phylogeny with LSD. Circles on branches represent variants that are compatible with the branch, i.e., they likely have emerged on that branch.
(TIF)

S8 Fig Evolutionary rates and date-randomization tests for the CAO. (A) Substitution rate and rates of randomized data for substitutions. (B) Short indel rate and rates of randomized data for short indels. Each grey dot is a rate estimate for a date randomization test, where the grey bars represent the associated confidence intervals.
(TIF)

S1 Table Samples and accession numbers. (A) Central Asian Outbreak. (B) Hamburg outbreak.
(XLSX)

S2 Table Antibiotic resistance-conferring genes in H37Rv. (XLSX)

S3 Table Number of variants in each category and raw numbers used for the enrichment analysis. The p-value is the result of Fisher’s exact test, with FDR-adjusted values.
(XLSX)

S4 Table Localization and description of the effect of parsimony informative indels. The column “Parsimony score” gives the minimum number of events required to explain the evolution of the variant on the phylogeny. Parsimony scores above 1 indicate incompatible variants. “Number of samples” describes the number of samples affected by the variant. “Effect” is either “IS6110 insertion”, “homopolymer” for an indel in a region with at least 3 repeated nucleotides, “in-frame” if the indel preserves the frame of the gene, “long deletion” for deletions longer than 50bp, or “other” for the remaining indels. “Position in gene” describes the localization of variants in coding regions by the gene coordinates. The essential category can be either essential (i.e., the gene is required for growth *in vitro*) or dispensable (i.e., the gene is not required for growth *in vitro*). “Antibiotic resistance” describes whether the gene has been found to confer antibiotic resistance upon substitutions (“Res”, according to S2 Table) or not (“NRes”). “Comment” describes whether a gene is partially or completely deleted in the case of long deletions or the genomic distance of intergenic variants from the neighboring genes. Intergenic variants are split in two rows, where the first row describes the gene before the variant, and the second row describes the gene after the variant, in genomic coordinates. Of note, four of the 10 incompatible short indels are found in homopolymer regions (positions 1,333,640bp, 4,140,029bp, 4,324,110bp and 4,359,787bp). In addition, four intergenic short indels that are incompatible likely stem from the same event, potentially involving a DNA translocation (positions 2,436,569bp, 2,436,573bp, 2,436,584bp and 2,436,585bp). The two remaining incompatible short indels constitute (i) an in-frame short deletion in a PE gene (position 1,212,205bp) and (ii) a 1bp insertion leading to a frameshift (and a modified protein sequence that is 28 amino acids longer), in a gene encoding a nitronate monooxygenase (position 3,082,251bp).
(XLSX)

S5 Table Description of incompatible SNPs. The column “Effect” gives the effect of the SNPs on the resulting protein, i.e., non-synonymous (NS) or synonymous (S). “Parsimony score” gives the number of independent events required to explain the evolution of the variant on the phylogeny. “Number of samples” describes the number of samples where the variant is detected. “Position in gene” describes the localization of variants in coding regions by the gene coordinates. The essential category can be either essential (i.e., the gene is required for growth *in vitro*) or dispensable (i.e., the gene is not required for growth *in vitro*). “Antibiotic resistance” describes whether the gene has been found to confer antibiotic resistance upon substitutions (“Res”, according to S2 Table) or not (“NRes”). “Comment” describes the genomic distance of intergenic variants from the neighboring genes. Intergenic variants are split in two rows, where the first row describes the gene before the variant, and the second row describes the gene after the variant, in genomic coordinates.
(XLSX)

S6 Table Summary of genes affected by convergent evolution due to indels. “Parsimony informative” states whether the variant considered is parsimony informative (Yes) or not (No). “Parsimony score” gives the minimum number of events required to explain the evolution of the variant on the phylogeny. “# SNPs in gene” gives the number of SNPs found in the gene considered. “Position in gene” describes the localization of variants in coding regions by the gene coordinates. The essential category can be either essential (i.e., the gene is required for growth *in vitro*) or dispensable (i.e., the gene is not required for growth *in vitro*). “Antibiotic resistance” describes whether the gene has been found to confer antibiotic resistance upon substitutions (“Res”, according to S2 Table) or not (“NRes”).
(XLSX)

S7 Table dN/dS analysis for genes having at least two SNPs. Genes are sorted by the number of SNPs they contain. “Antibiotic resistance” describes whether the gene has been found to confer antibiotic resistance upon substitutions (“Res”, according to S2 Table) or not (“NRes”). The essential category can be either essential (i.e., the gene is required for growth *in vitro*) or dispensable (i.e., the gene is not required for growth *in vitro*).
(XLSX)

S8 Table Command lines and parameters for variant calling, regenotyping and reads simulation. (XLSX)
