# Supplementary figures and images for: Insertion and deletion evolution reflects antibiotics selection pressure in a Mycobacterium tuberculosis outbreak
Source: PLoS Pathog. 2020 Sep 30;16(9):e1008357. doi: 10.1371/journal.ppat.1008357 (PMC7549793; doi:10.1371/journal.ppat.1008357)

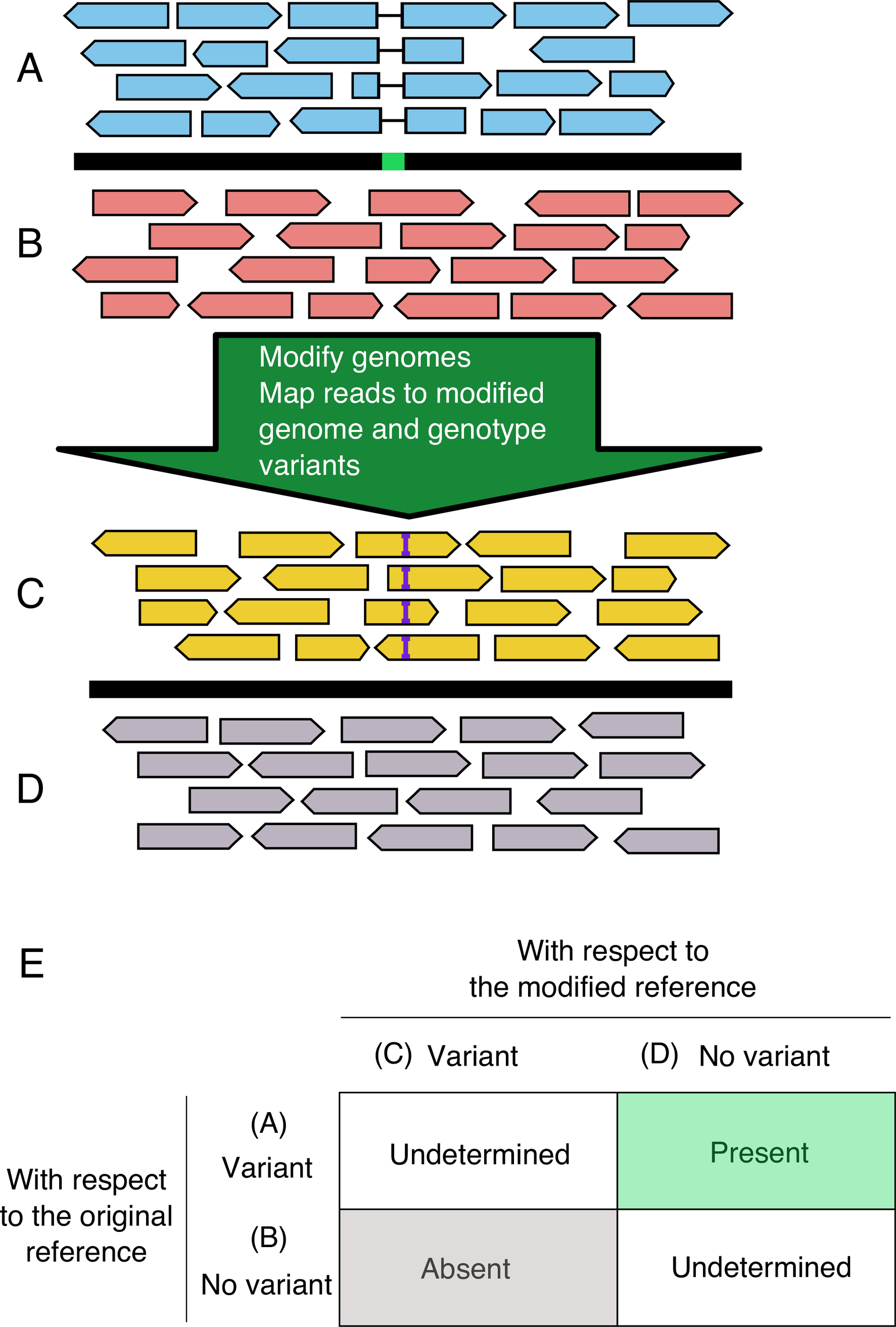

Supplement: S1 Fig — The example shows the calling and back-genotyping of a small deletion. (A)-(B) We identify a short deletion in one sample (blue). We include this deletion in the modified genome and map all the samples, and we genotype variants. (C)-(D) We either detect an insertion (yellow sample), if the base pairs that are removed in the modified genome are present in the reads, or nothing if the deleted portion is not present in the reads (grey sample). (E) The decision table summarizes the decision on the variants for each sample. If a variant is detected in the first phase and the inverse variant is not detected in the second phase, the variant is considered present. Alternatively, if the variant is not detected in the first phase but the inverse variant is detected in the second phase, the variant is considered absent. For the remaining cases, the variant is considered undetermined. These decisions are summarized in a presence-absence matrix, where the variants are denoted as present (1), absent (0), or undetermined (gap, “-“). (TIF) [file ppat.1008357.s001.tif]

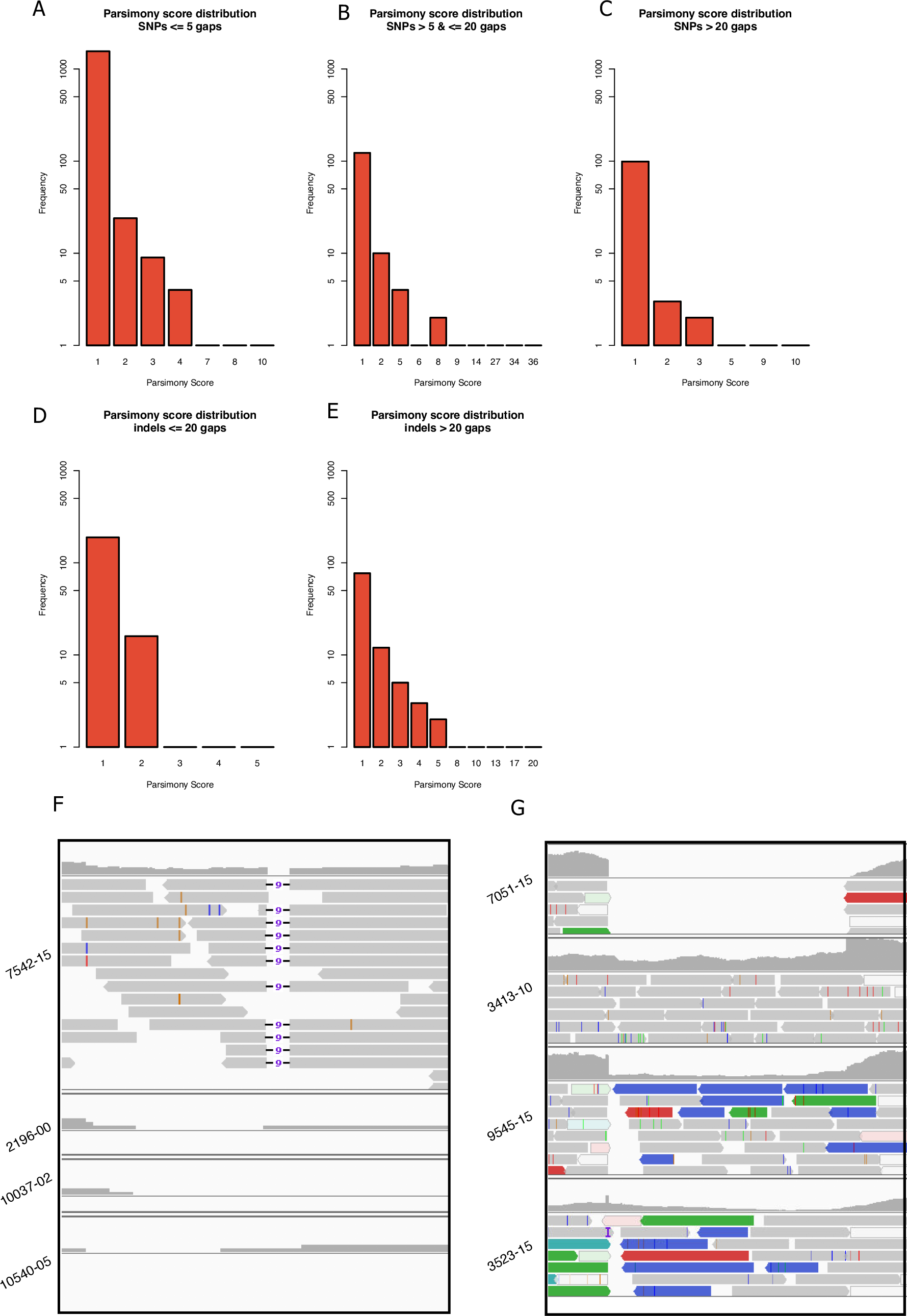

Supplement: S2 Fig — (A) Parsimony scores of SNPs having at most five gaps (i.e., SNPs that are undetermined in at most five samples), (B) Parsimony scores of SNPs having between six and 20 gaps. (C) Parsimony scores of SNPs having more than 20 gaps, (D) Parsimony scores of indels having at most 20 gaps, (E) Parsimony scores of indels having more than 20 gaps. The parsimony scores of SNPs between six and 20 gaps are high (up to 36), therefore we excluded SNPs having more than five gaps. Indels exhibit rather low parsimony scores (one to five) for variants exhibiting at most 20 gaps. Variants having more than 20 gaps have higher parsimony scores (up to 20), therefore the threshold has been set to 20 gaps for including indels. (F) Example of a deletion of 9bp that is undetermined in some samples displayed with IGV. This deletion was detected as present in seven samples (here 7542–15) and undetermined in 118 samples (here 2196–00, 10037–02 and 10540–05), hence it was filtered out according to our criteria. This example illustrates the importance of back-genotyping for real sequencing data in genomic regions that are difficult to align. Here, the coverage in the samples where the deletion was not detected is nearly zero, thereby not allowing for a confident variant inference. (G) Example of a deletion of 438bp that is undetermined in some samples displayed with IGV. This deletion was detected as present in 133 samples (here 7051–15) absent in two samples (here 3413–10) and undetermined in 218 samples (here 9545–15 and 3523–15). In samples where the deletion is undetermined, while we see a decrease of coverage potentially associated with a deletion, the reads tend to show rearrangements (i.e., displayed as different read colors in IGV). Assigning absences would have potentially led to a misleading phylogenetic reconstruction since we cannot ascertain the presence or absence of these variants in the low-covered samples or samples with putative rearrangements. (TIF) [file ppat.1008357.s002.tif]

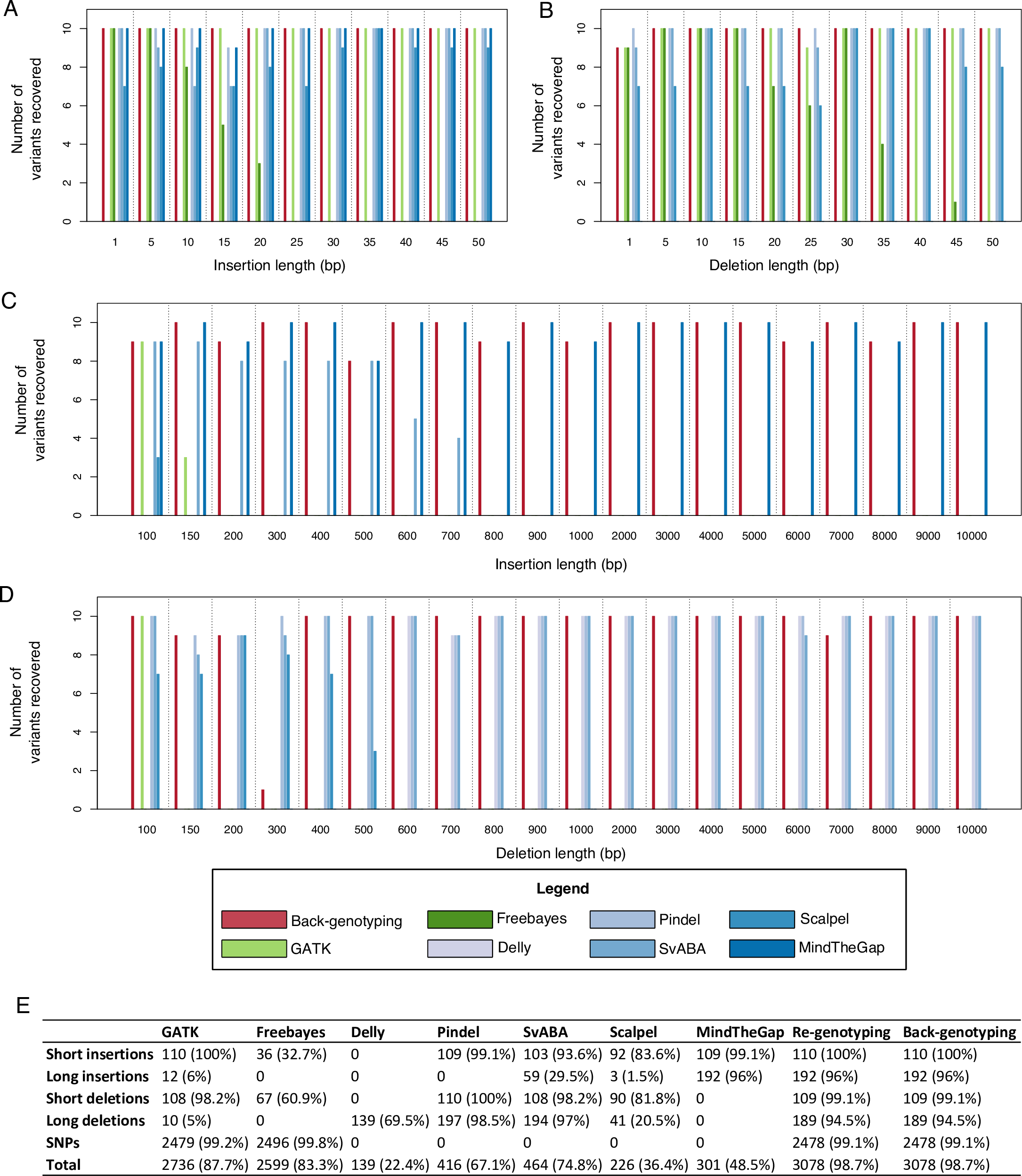

Supplement: S3 Fig — Ten replicates have been carried out for each setting. (A) Short insertions, (B) short deletions, (C) long insertions, (D) long deletions, (E) table of variants found by each step of the variant calling. The combination of the variants detected by each variant calling tool allowed to retrieve a total of 3,104 variants (99.5%). In addition, we found 157 false positives (17 SNPs and 140 indels up to 77bp) with GATK and Freebayes when calling long indels (>50bp). Since these tools are not designed to find such long variants, false positives could occur due to misalignment of the reads in missing or added portions of the genome. After re-genotyping, there are 3,078 true variants (600 indels, 96.8% of all indels) and 144 false positives. All these variants were also back-genotyped. The high sensitivity of the approach in simulations reflects, in part, that the variants were included in the same genomic backbone that was used as a reference genome. (TIF) [file ppat.1008357.s003.tif]

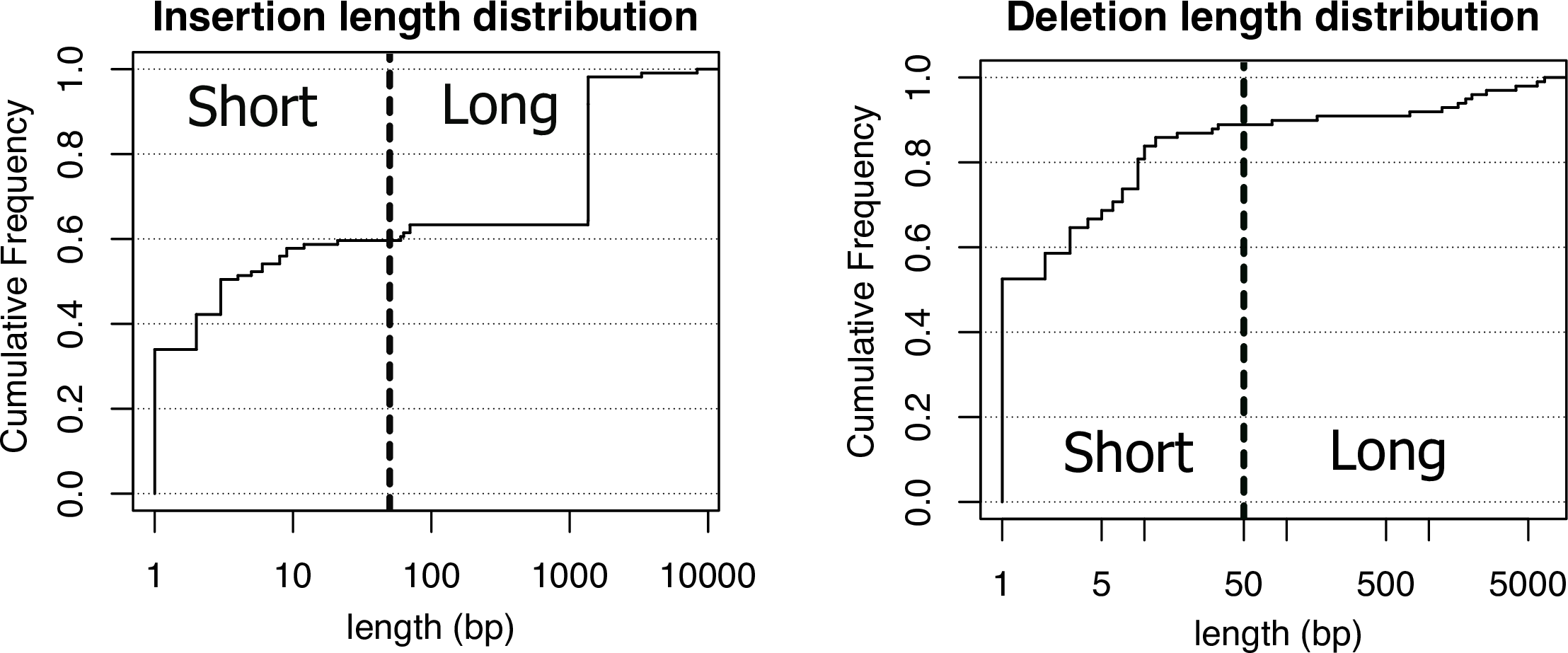

Supplement: S4 Fig — 65 of 44 insertions are short (59.6%) and 88 of 99 deletions are short (88.9%). (TIF) [file ppat.1008357.s004.tif]

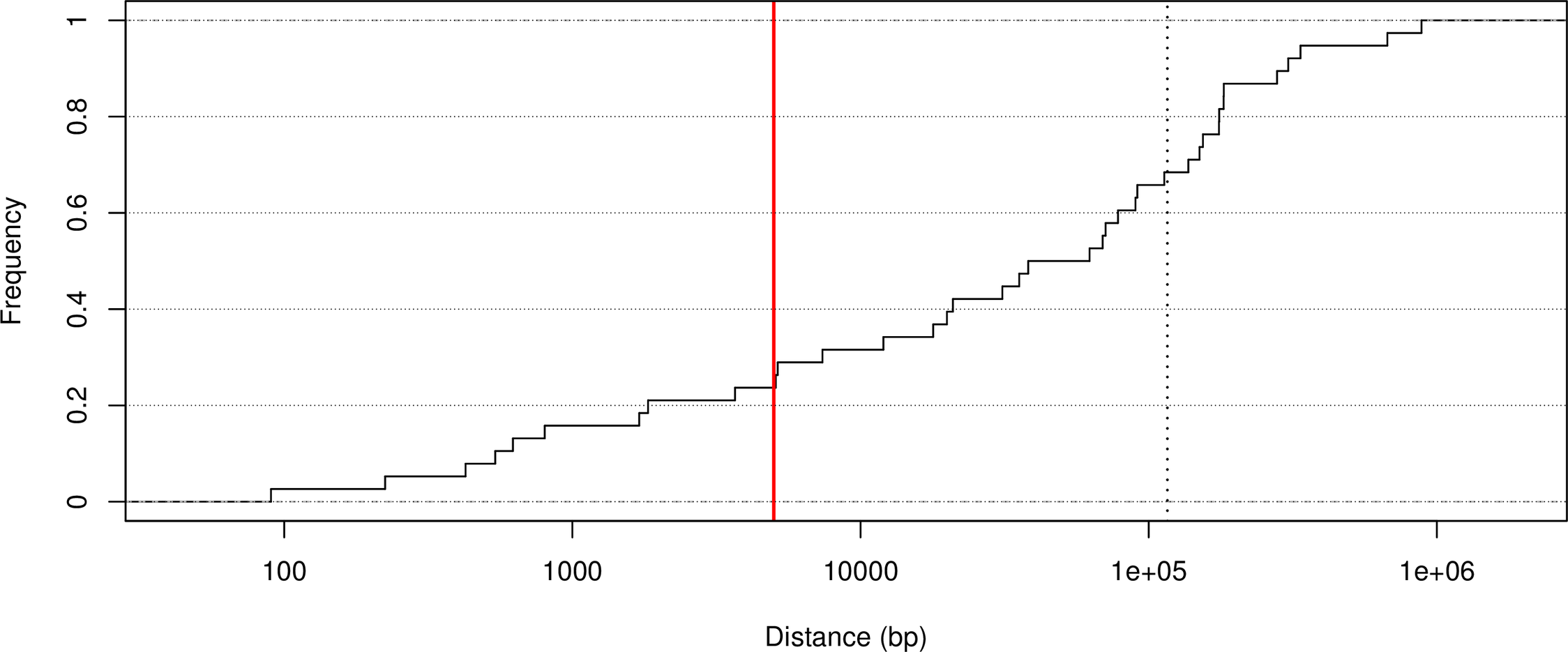

Supplement: S5 Fig — The grey dotted line shows the expected distance between neighboring IS if they were uniformly distributed along the genome (116,115 bp). Here, we defined an IS6110 insertional hotspot as at least two insertions, distant of at most 5,000 base pairs (red line), in two different samples. The distance cutoff was chosen since it includes ten of 38 neighboring pairs (26.3%). In our data, we could classify 17 of the 38 IS insertions (44.7%) into eight hotspots. Two of the hotspots have already been reported and they correspond to insertions into the DnaA-DnaE intergenic region (region 3013bp– 3237bp in the genome) and the phospholipase C region (2,623,208bp– 2,625,716bp). The six remaining hotspots are located in genomic regions consisting mainly of hypothetical proteins (regions in the genomes are: 2,030,702bp– 2,031,324bp, 2,551,089bp– 2,554,757bp, 2,574,702bp– 2,575,128bp, 2,610,662bp– 2,610,752bp, 3,481,063bp– 3,482,894bp and 3,545,241bp– 3,545,781bp). (TIF) [file ppat.1008357.s005.tif]

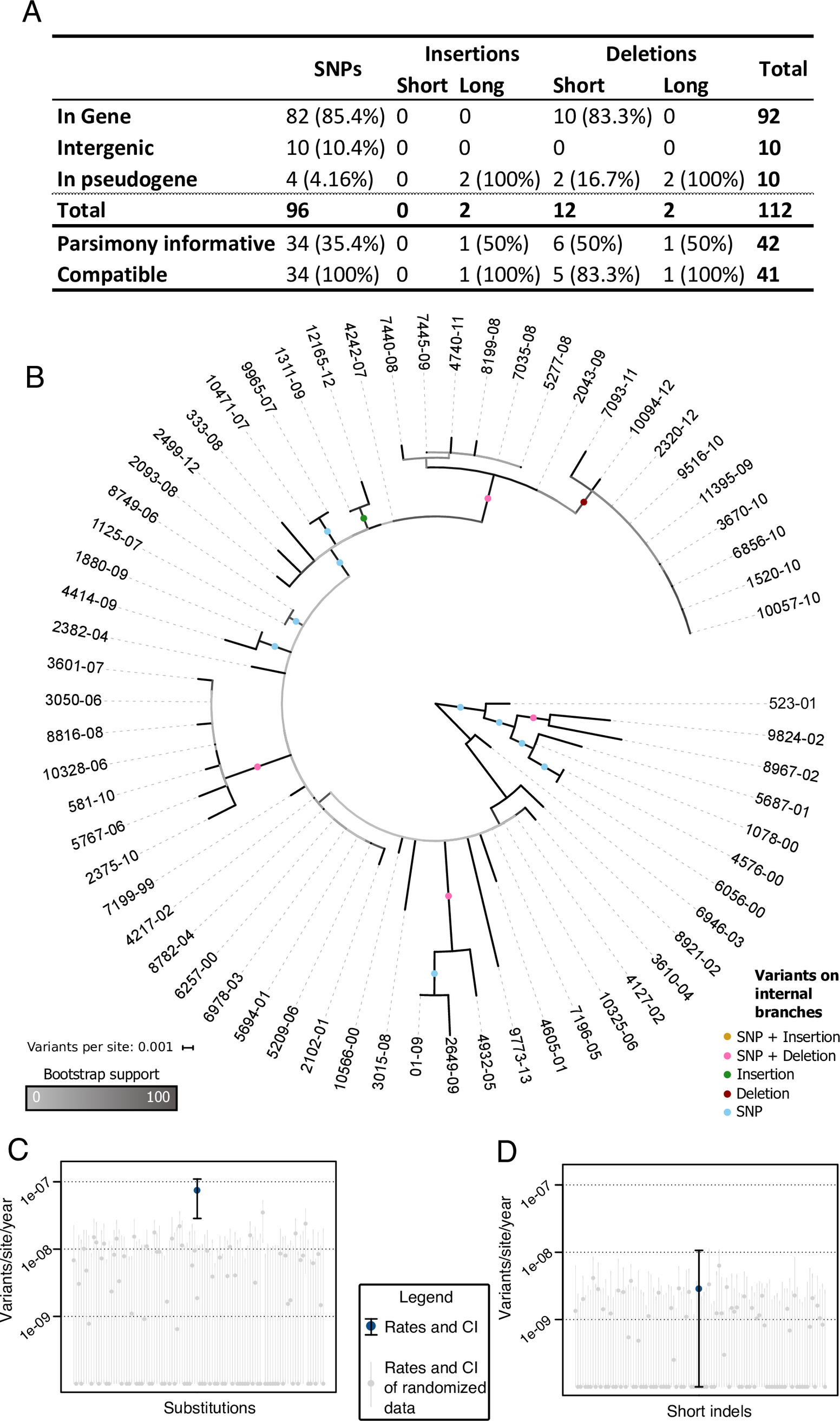

Supplement: S6 Fig — The Hamburg outbreak is a fully-sensitive MTB strain of lineage 4 sampled in the Hamburg and Schleswig-Holstein region between 1998 and 2013 (S1B Table; Roetzer et al., 2013 [28]). (A) Summary and genomic localization of detected variants. Percentages were calculated based on the total number of variants in each variant class, except for the compatible variants, where the percentage was calculated based on the number of parsimony informative variants in each variant class. The two long insertions correspond to IS6110 insertions, inferred as two alternative insertions in a pseudogene annotated as a Fic protein. No variants were found in ABR-conferring genes and the enrichment analysis on the essential category did not show any significant results. (B) Phylogenetic tree of the Hamburg outbreak, inferred from the presence-absence patterns of 112 detected variants and rooted by the temporal root estimated with LSD. Our approach found that all variants but one deletion are compatible with the phylogeny. Notably, two branches are refined by single indels. One is an IS6110 insertion that groups two samples, the second is a 45bp deletion in a PE gene that groups nine samples. (C) Substitution rate and 95% confidence intervals (CI) estimated with LSD. Substitutions have temporal signal according with the intermediate test for temporal signal, with an estimated substitution rate of 7.51e-8 [2.85e-8–11.0e-8] substitutions/site/year, where the confidence interval (CI) includes the previous estimate of 1e-7 substitutions/site/year (Roetzer et al., 2013 [28]). (D) Short indel rate and 95% confidence intervals. The overlap between the rates of the date-randomized data shows that there is not sufficient temporal signal to estimate evolutionary rates for indels. The absence of temporal signal might be traced back to the low number of indels in this data set. (TIF) [file ppat.1008357.s006.tif]

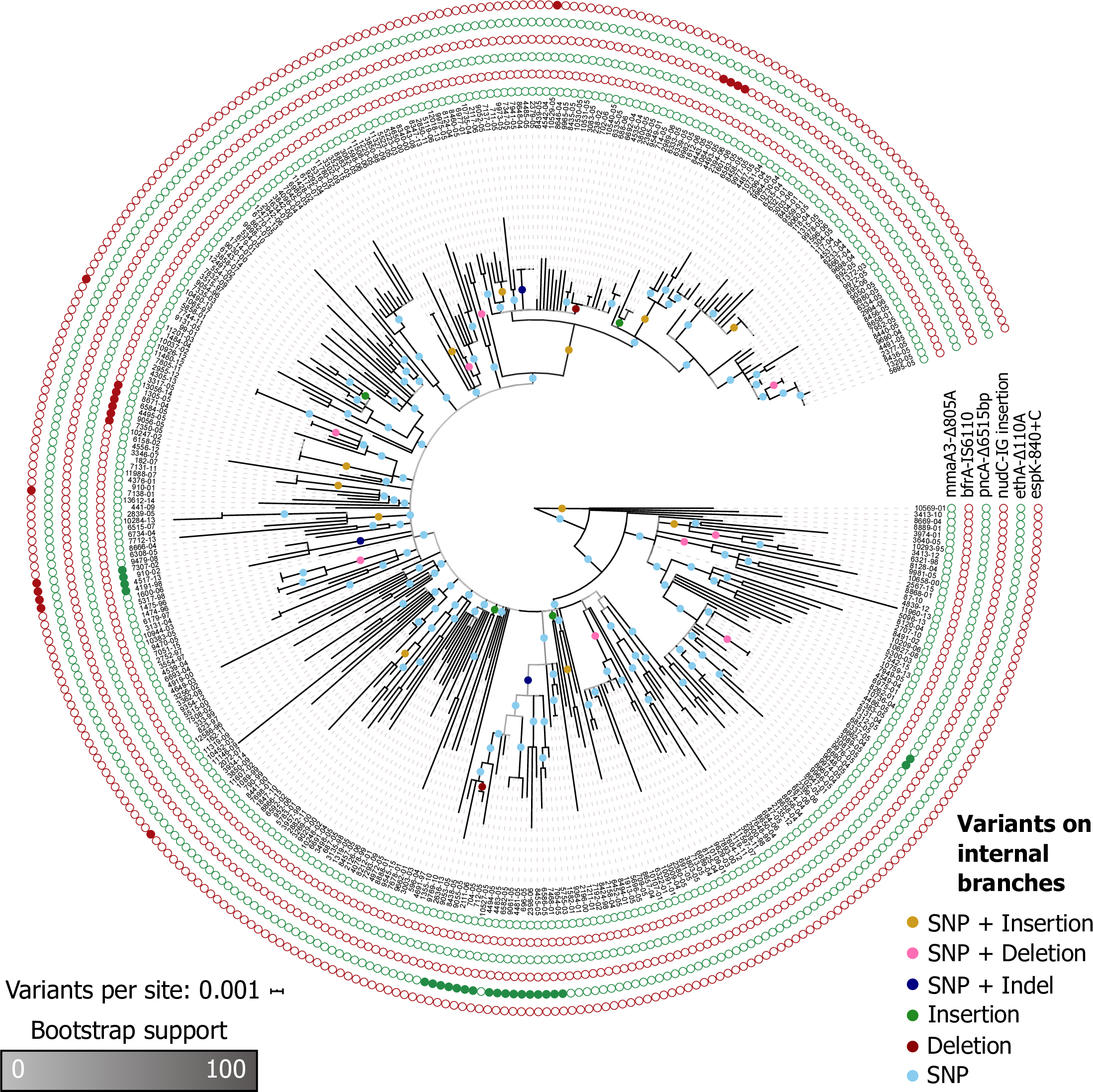

Supplement: S7 Fig — The root position is the temporal root estimated by dating the phylogeny with LSD. Circles on branches represent variants that are compatible with the branch, i.e., they likely have emerged on that branch. (TIF) [file ppat.1008357.s007.tif]

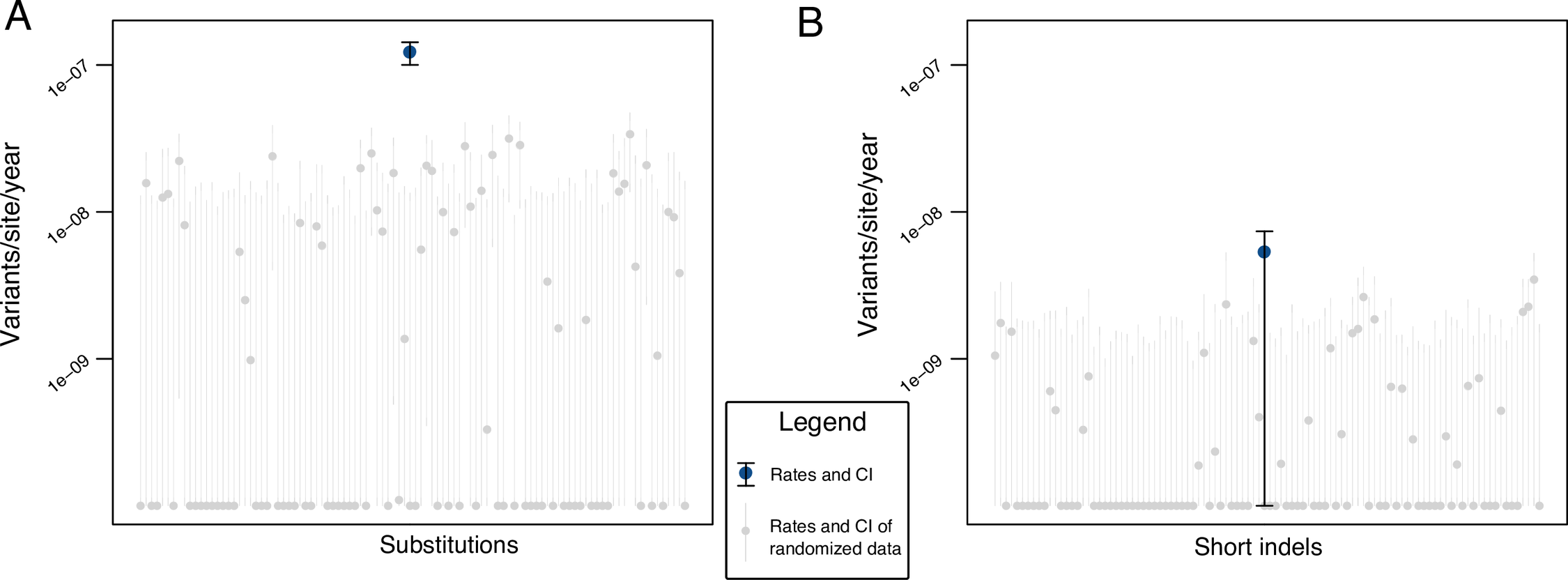

Supplement: S8 Fig — (A) Substitution rate and rates of randomized data for substitutions. (B) Short indel rate and rates of randomized data for short indels. Each grey dot is a rate estimate for a date randomization test, where the grey bars represent the associated confidence intervals. (TIF) [file ppat.1008357.s008.tif]
